# malERA: An updated research agenda for diagnostics, drugs, vaccines, and vector control in malaria elimination and eradication

**DOI:** 10.1371/journal.pmed.1002455

**Published:** 2017-11-30

**Authors:** The malERA Refresh Consultative Panel on Tools for Malaria Elimination

## Abstract

Since the turn of the century, a remarkable expansion has been achieved in the range and effectiveness of products and strategies available to prevent, treat, and control malaria, including advances in diagnostics, drugs, vaccines, and vector control. These advances have once again put malaria elimination on the agenda. However, it is clear that even with the means available today, malaria control and elimination pose a formidable challenge in many settings. Thus, currently available resources must be used more effectively, and new products and approaches likely to achieve these goals must be developed. This paper considers tools (both those available and others that may be required) to achieve and maintain malaria elimination. New diagnostics are needed to direct treatment and detect transmission potential; new drugs and vaccines to overcome existing resistance and protect against clinical and severe disease, as well as block transmission and prevent relapses; and new vector control measures to overcome insecticide resistance and more powerfully interrupt transmission. It is also essential that strategies for combining new and existing approaches are developed for different settings to maximise their longevity and effectiveness in areas with continuing transmission and receptivity. For areas where local elimination has been recently achieved, understanding which measures are needed to maintain elimination is necessary to prevent rebound and the reestablishment of transmission. This becomes increasingly important as more countries move towards elimination.

## Abstract 2

David Kaslow and colleagues examine the progress in reasearch for diagnostics, drugs, vaccines, and vector control in malaria elimination and eradication and propose a research agenda.

**Summary points**

- Achieving malaria elimination likely requires new interventions and strategies in some settings. In addition, the effectiveness of existing tools must be preserved and tools deployed to counter the numerous challenges, key among which are the emergence and spread of drug-resistant parasites and mosquitoes with resistance to vector control measures.
- The key research goal for diagnostics is the detection of populations with subclinical infections and low parasite counts. Such diagnostics enable the development of effective surveillance systems directed at malaria parasite elimination.
- The availability of new transmission-blocking drugs, vaccines, and vector control products would accelerate elimination where there is refractoriness to currently available interventions. New regulatory pathways and product development models are needed to efficiently develop and assess these new interventions.
- In areas endemic for *Plasmodium vivax* and *P*. *ovale*, the hypnozoite reservoir must be targeted with more robust tools and strategies.
- In areas of declining transmission, as cases become less frequent, the contribution to transmission of the subclinical parasite reservoir needs to be quantified and addressed with transmission-blocking interventions.
- For vector control, addressing continuing escalation of insecticide resistance—including through the identification of new chemical classes and longer-lasting insecticide formulations—remains a priority. Changes in vector populations and behaviours must also be addressed to restore responsiveness to existing interventions. In some areas, new paradigms may be needed to understand how to design interventions that reduce vector populations and receptivity to sufficiently low levels.
- Policy and decision makers, faced with chronic resource limitations, insufficient surveillance, spatial and temporal heterogeneity of malaria parasite transmission, and multiple intervention choices, need improved strategies and guidance on how, where, and when to best combine and deploy existing and new interventions to maximise their longevity and effectiveness.

## Introduction

Achieving malaria parasite elimination across all countries (i.e., malaria eradication), especially for those with a high disease burden, likely requires new tools and strategies to complement existing interventions [1,2]. Given the inevitable uncertainties in product development and given that different sets of tools will be applicable in different settings, a broad and imaginative research and development agenda needs to be pursued. The research and development agenda presented in this paper is in support of the WHO Global Technical Strategy for malaria goals from 2016 to 2030, and tracking the progress of this research and development (R&D) agenda and reevaluating the research needs will be required over time [2]. In Malaria Eradication Research Agenda (malERA) 2011, diagnostics, drugs, vaccines, and vector control were considered separately [3–6]. However, for malERA Refresh, this paper considers together the research agenda for all existing and prospective tools to accelerate progress towards achieving and maintaining malaria elimination. In this case, the relationships between the different research agendas can be more easily recognised. Other papers in this malERA Refresh series consider the related discussions regarding the implementation and combination of tools [7], implications of insecticide and antimalarial drug resistance [8], health system and policy issues [9], and advances in basic science [10].

### Progress on tools for malaria elimination

Based on literature reviews and panel consultations [11], the most significant advances in the development and deployment of malaria control and elimination tools between 2011 and 2015 were identified (S1–S4 Texts). For diagnostics, advances include widespread incorporation of *P*. *falciparum* rapid diagnostic tests (RDTs) into routine malaria case management [12], development of highly sensitive tools for detecting subclinical infections, and development and deployment of combined tests that differentiate *P*. *falciparum* from *P*. *vivax* [13,14]. For drugs, advances include the deployment of hundreds of millions of artemisinin-based combination therapy (ACT) courses [12], publication of guidelines for mass drug administration (MDA) [15], the recommendation of low-dose primaquine for transmission interruption [16], progression of new antimalarial compound classes into clinical development [17–19], field trials to evaluate the potential role of medicines in killing mosquitoes (endectocides) [2], and the identification of *Kelch13* as a marker for artemisinin resistance, enabling mapping of its geographic distribution [20,21]. For vaccines, advances include the Article 58 positive opinion by the European Medicines Agency and recommendations by the World Health Organization (WHO) on the first vaccine targeting malaria, RTS,S-AS01_E_ (Box 1) [22–33]; revision of the Malaria Vaccine Technology Roadmap [34]; and new vaccines that progressed to clinical trials [35,36]. For vector control, advances include registration of 2 additional long-lasting insecticide formulations for indoor residual spraying (IRS) [37,38], field trials of dual-insecticide bed nets [39–41], development programmes for new insecticides [42–44], and publication of the larval source management operational manual by WHO [45]. Advances have also been made in the ‘tools for developing tools’—for example, controlled human malaria infection (CHMI) blood-stage parasite inoculation (Box 2) [46–56]; the human blood-stage challenge model for early-stage determination of antimalarial drug pharmacokinetics/pharmacodynamics [57]; the development of human liver chimeric mice, human erythroid chimeric mice, and dually engrafted mice allowing replication of the entire *P*. *falciparum* life cycle [58]; and validation of phenotypic assays for gametocyte screening to identify compounds with transmission-blocking activity [59]. In addition, new technologies and scientific insights are emerging [10], with notable improvements in mapping and modelling [7,60–62].

**Box 1. Malaria vaccine RTS,S/A101_E_**

In 2015, the preerythrocytic candidate vaccine RTS,S/AS01_E_ (RTS,S) received a positive scientific opinion by European regulators through the Article 58 procedure. This was a breakthrough in malaria vaccine development, identifying a regulatory pathway and demonstrating that the large clinical trials necessary for approval could be conducted in Africa [23–25].

- The target for RTS,S is the reduction of malaria incidence and severe disease in young children. A 3-dose regimen was shown to reduce the number of malaria cases by half in children 5–17 months of age during the first year following vaccination; efficacy waned over time but was prolonged by a fourth dose [25].
- Despite modest efficacy, RTS,S prevented about 1,700 cases for every 1,000 children vaccinated in a phase III study over a 4-year period, and modelling studies predict a considerable public health impact for RTS,S, with the greatest benefit expected in areas with the highest malaria burden [27].
- Following review of RTS,S data by the Strategic Advisory Group of Experts on Immunization and the Malaria Policy Advisory Committee, in 2016 the WHO adopted recommendations for RTS,S pilot implementations in 3–5 settings involving 100,000–200,000 children per setting (for a total of 400,000–800,000), in a staged manner to further evaluate safety (including meningitis [26]), feasibility of delivery, and impact on mortality.
- Phase IV studies with a primary objective of further evaluation of safety as part of the Risk Management Plan approved by European Medicines Agency are planned to be linked to the larger pilots, with complementary design and objectives [28,29].

**Research to optimize the regimen and explore additional applications of RTS**,**S**

○ **Optimising the RTS,S dosing regimen**
  Additional controlled human malaria infection (CHMI) and phase IIb studies are in progress to better define how to improve RTS,S/AS01 efficacy and how these data translate to the field, respectively.
  - A small study with RTS,S and an earlier adjuvant (AS02) found that fractional dosing, i.e., 2 full monthly doses plus a third low-dose at 7 months, resulted in apparent high efficacy against *P*. *falciparum* challenge (6/7 protected) [30].
  - A recent CHMI study in a larger number of volunteers using RTS,S/AS01 confirmed that a 0-, 1-, 7-month regimen that included a fractional third dose (Fx017M) was associated with higher efficacy (86.7% [95% confidence interval [CI], 66.8%–94.6%]; 26/30 protected) than the standard monthly full-dose regimen (62.5% [95% CI, 29.4%–80.1%]; 10/16 protected) against infection 3 weeks after the third dose [31].
○ **Additional applications for RTS,S**
  Additional applications of RTS,S explored through modelling and, if indicated, evaluated in carefully designed field studies over the next 5-year period include the following [33]:
  - Evaluating the contribution to elimination of artemisinin-resistant parasites in the Greater Mekong Subregion, although data supporting an adult indication (dose and regimen) would be needed [32];
  - Combining RTS,S with other interventions or another malaria vaccine (mass drug administration [MDA] or a future VIMT, respectively), with the aim of enhancing or extending their effects;
  - Combining RTS,S with seasonal malaria chemoprevention (SMC), a study of which is in progress in Burkina Faso and Mali.

**Box 2. Tools for developing tools: Demonstrating transmission-blocking activity**

The validation of surrogate end points for transmission-blocking activity that translate into known effects in the field is necessary for the efficient development of new interventions aimed at this target.

**Mosquito** **feeding assays**

Three assays are available for assessing transmission-blocking activity:

- Direct feeding assay (DFA): allowing mosquitos to feed on parasitaemic hosts; the most ‘physiologically relevant’ method [46,47].
- Direct membrane feeding assay (DMFA): blood samples from parasitaemic hosts are fed to susceptible mosquitoes through an artificial membrane [48].
- Standard membrane feeding assay (SMFA): laboratory-reared mosquitoes are fed a controlled number of cultured gametocytes from a single parasite strain combined with uninfected human erythrocytes and serum from human volunteers or animals. SFMA is now available as a medium throughput, reproducible, standardised assay [49].

In the context of elimination, the relevant outcome from these assays is a reduction in the number of infected mosquitoes.

**Controlled human malaria infection** **(CHMI) model**

Three CHMI techniques have been developed to determine the ability of drugs and vaccines to prevent human infection:

- Sporozoite mosquito bites: infection of human volunteers via mosquito biting [50,51].
- Sporozoite direct venous inoculation (SDVI): injection of sporozoites into human volunteers [52,53].
- Induced blood-stage malaria parasite infection (IBSM): administration of *Plasmodium*-infected red blood cells to human volunteers [54,55].

Each of these techniques has advantages and disadvantages. Both sporozoite-based models allow evaluation of preerythrocytic and blood-stage drugs and vaccines, whereas IBSM can determine blood-stage efficacy only.

To evaluate transmission-blocking efficacy in preventing transmission from humans to mosquitoes, CHMI can be followed by a mosquito feeding assay using blood or serum from CHMI volunteers. Development of a regulatory pathway using mosquito feeding assays and CHMI with relevance to transmission-blocking activity in the field is ongoing. This effort would benefit from the development of new vaccines and drugs aimed specifically at this indication [56].

## Diagnostics research agenda

### Diagnostics for malaria treatment and elimination

To direct malaria treatment, all cases should be confirmed with a diagnostic test, either RDT or light microscopy, even in low-transmission settings [63]. Current WHO criteria for RDT procurement recommend a false positive rate of <10% [14]. However, a test with 99% specificity, when used at the elimination threshold (prevalence of parasitaemia in the community of ≤0.1%), results in ≥90% of positive tests coming from samples with no *Plasmodium* parasites [64]. In very low-transmission settings, addressing the challenge of false positive tests may require developing algorithms such as parallel or serial confirmation with a second test. Recently, false-negative results for *P*. *falciparum* histidine-rich protein 2 (PfHRP2)-based RDTs have been reported from several regions, caused by pfhrp2/pfhrp3 gene deletions [65–70]. Universal validity of these diagnostic tests cannot be assumed, and the WHO has issued guidance on PfHRP2-based RDT procurement [71].

Beyond *P*. *falciparum*, improved RDTs are needed for other species. Available lactate dehydrogenase (LDH)-targeting RDTs are less sensitive for *P*. *vivax* compared to *P*. *falciparum*, because *P*. *vivax* parasite densities tend to be much lower [72]. There is a paucity of information on test performance against minor species.

**Fig 1:**
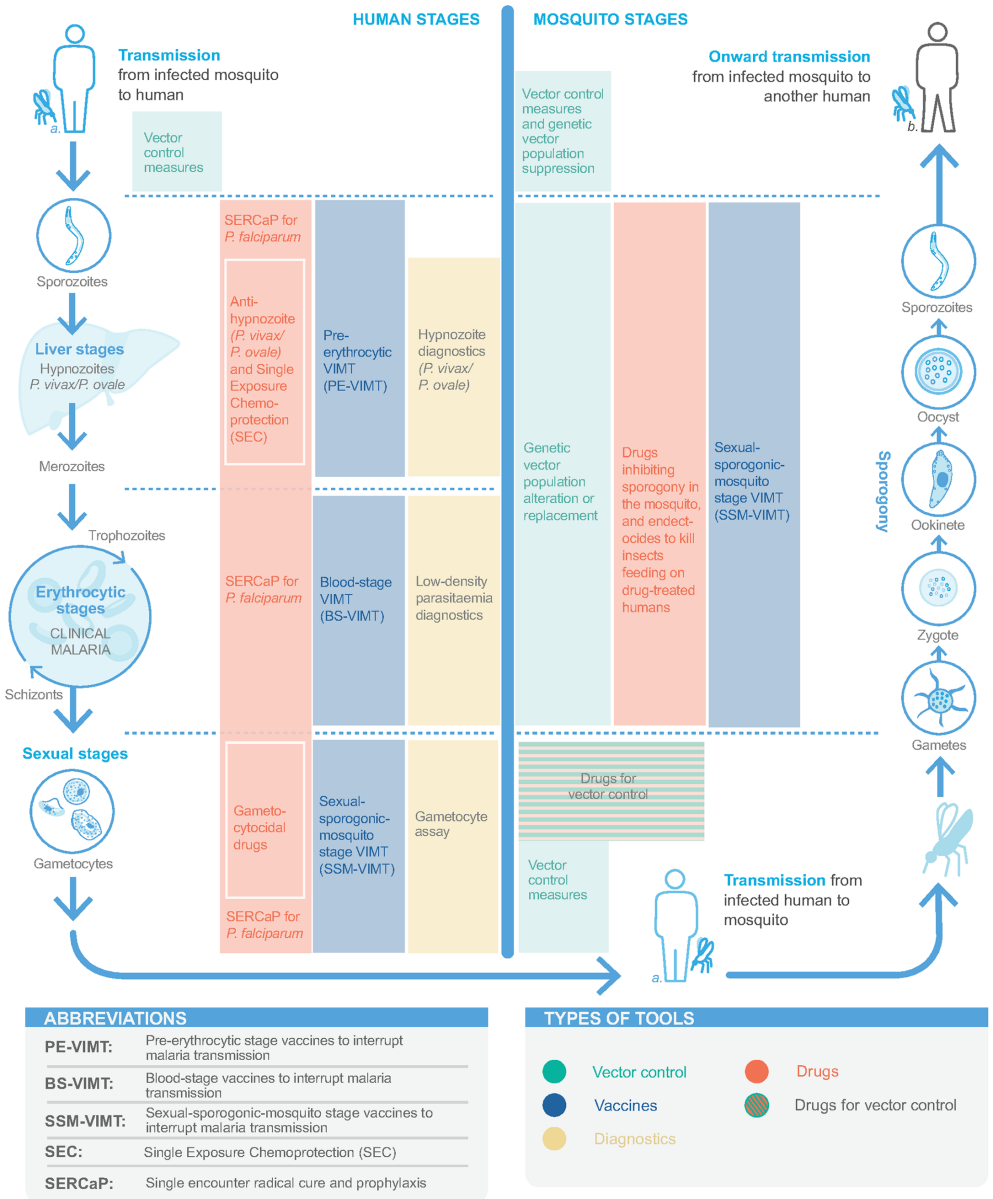
Tools for detecting and interrupting malaria transmission and their action in the malaria transmission cycle.

In the elimination context, a malaria diagnostic tool is needed for reactive or proactive detection of infectious parasite reservoirs residing in those individuals with subclinical infections and/or with parasite densities lower than those reliably detected with existing RDTs and microscopy (Fig 1) [73]. A target product profile (TPP) has been developed for a point-of-care malaria infection detection test for rapid detection of low-density, subclinical malaria infections [64]. Provided this was sufficiently sensitive, it would potentially enable targeting of populations harbouring reservoirs of parasite biomass with interventions interrupting transmission.

The most efficient uses for digital health are still being explored in malaria, but integrating diagnostic results generated from malaria case management into elimination programme surveillance efforts offer a near-term opportunity to fill critical data gaps in mapping malaria prevalence [2,7]. For example, 1 study combined a globally accessible database with mobile phone-based imaging of RDTs to provide an objective diagnostic readout and automated collection of surveillance data [74]. A similar approach in Kenya used digital RDT readers with upload to a cloud database [75]. However, lessons learned from digital health applied to the eradication programme for tuberculosis suggest that attaining a population-level impact are undermined by insufficient scale, coordination, and end-user engagement [76]. These issues are likely compounded in malaria given the higher prevalence of the disease globally.

#### Approaches to developing diagnostics

Several biomedical engineering approaches for malaria parasite detection have been investigated [77], including automated image processing [78], microfluidic systems [79], microarray chips [80], dielectrophoresis [81], and exploiting the bioelectrical properties of blood [77]. Further development of these techniques to increase sensitivity and specificity to detect clinically unapparent malaria parasite infections and allow field deployment continues.

Simplified molecular methods to detect low-level *P*. *falciparum* parasitaemia for use in low-resource settings are being developed, although improvements in throughput and cost are required [82]. Loop-mediated isothermal amplification (LAMP) is 1 promising approach, already validated in low-transmission settings [83] and as point-of-care detection of asymptomatic low-density malaria parasite carriers [84]. Further developments include noninstrumented nucleic acid amplification LAMP (NINA-LAMP) [85], achieving comparable sensitivity to *P*. *falciparum* polymerase chain reaction (PCR) detection in the field [85,86]. Another approach, using an insulated isothermal PCR (iiPCR) in a commercially available portable device, for *Plasmodium* detection achieved an assay efficiency of 96.9% with a lower detection limit of ≥100 copies of plasmodial DNA [87]. Nucleic acid amplification techniques can also be used for multiple pathogens in parallel, incorporating other infectious diseases (e.g., Ebola, dengue, and typhoid), depending on the setting and target population [88].

### Noninvasive testing

Although a noninvasive technique is highly preferred, all currently available diagnostics require blood samples. PCR-based assays to detect *Plasmodium* parasites in saliva, although unsuitable for routine diagnosis, have been successfully developed [89]. Malaria detection in urine has been evaluated in field trials, but its sensitivity requires improvement [90]. Preliminary investigations indicate that malaria-specific volatile levels from breath samples correlate with parasite clearance [91], and studies are ongoing. A transdermal, noninvasive, reagent-free approach relying on the presence of iron-rich haemozoin to generate vapour nanobubbles is currently being field tested to detect parasites in skin blood vessels [92].

### Detecting gametocytes

While gametocyte detection may indicate an individual’s transmission potential, further definition is required as to the most appropriate clinical sample to collect and the relevant gametocyte levels reflecting infectiousness [47]; this is complicated by a lack of correlation between gametocyte density in the blood and infectiousness following antimalarial treatment [93]. Validation of relevant target sequences is a first step towards development of molecular methods amenable for routine gametocyte detection. Circulating *P*. *falciparum* female and male and *P*. *vivax* gametocytes can now be detected using quantitative nucleic acid sequence-based amplification (QT-NASBA) or quantitative PCR (qPCR) methods with pfs25-, pfs230p-, and pvs25-based primers, respectively [94–98].

### Detecting drug resistance

Detection of Kelch-propeller polymorphisms conferring artemisinin-resistance is currently restricted to sentinel surveillance [21], though more granular information is needed with continuing efforts to eliminate artemisinin-resistant parasites [99]. For example, a next-generation amplicon sequencing method suitable for use in endemic countries enables high-throughput detection of genetic mutations in 6 *P*. *falciparum* genes associated with resistance to antimalarial drugs, including artemisinins, chloroquine, and sulfadoxine-pyrimethamine [100]. For detecting *P*. *falciparum* single nucleotide polymorphisms (SNPs) associated with antifolate drug resistance, the ligase detection reaction fluorescent microsphere (LDR-FM) assay has been validated in clinical trials in Uganda [101]. As noted elsewhere in the malERA Refresh series [8], continued research on identifying markers of resistance to the other antimalarial drugs in current use (e.g., lumefantrine and piperaquine) is critical, as tools are needed to detect and manage drug resistance inevitable in elimination efforts [102,103].

### Detecting hypnozoites

Hypnozoites residing in the human host is one tactic used by *P*. *vivax* and *P*. *ovale* to sustain the parasite reservoir between transmission seasons and produce multiple clinical relapses over prolonged periods, each with the potential to maintain transmission [104]. Detecting *P*. *vivax*/*P*. *ovale* hypnozoites, however, is problematic because of their low density, metabolic inactivity, and sequestration within the liver. Biomarkers that detect hypnozoites would be breakthrough tools in both case management diagnostics and elimination surveillance for *P*. *vivax* and *P*. *ovale* infections.

### Glucose-6-phosphate dehydrogenase (G6PD) testing

An affordable, easy-to-use, rapid, point-of-care, semiquantitative diagnostic test is needed to identify G6PD-deficient individuals at risk of haemolysis with use of 8-aminoquinolones (i.e., primaquine or tafenoquine) to prevent *P*. *vivax* relapses. Although several tests are available [105], further refinement is needed to support greater access to these medicines. Single administration of low-dose (0.25 mg/kg) primaquine as a gametocytocidal agent is recommended after treatment for *P*. *falciparum* malaria [106], but there is still a need for more data on the optimal dose and reassurance of safety in G6PD-deficient individuals and larger populations, if used in MDA, for example [107].

### Pregnancy testing

Pregnant women are excluded from receiving certain drugs or interventions, and rapid, low-cost, low-complexity, point-of-use pregnancy tests are needed, particularly for populations receiving MDA drugs with contraindications for use during pregnancy.

### Challenges

Diagnostics are needed to direct treatment, support surveillance, and identify transmission reservoirs [73] and for continued progress in the development and evaluation of other tools for elimination, e.g., in settings with low-density parasitaemia and low transmission and for interventions targeting hypnozoites/prevention of relapse. The relevance of low-density parasitaemia to transmission requires further investigation to enable the design of diagnostics appropriate for these needs. Longer term, development of noninvasive assays, and field assays for detecting drug-resistant parasites should be pursued. Detecting hypnozoites remains a more profound challenge, although proteomics and metabolomics are being explored [108].

## Drug research agenda

### Drugs for malaria treatment, prevention, and transmission interruption

A strong portfolio of combination medicines with different or competing resistance mechanisms is required to combat resistance. It is now possible to tune the development program to advance drugs that have high barriers to resistance development and a low potential for cross resistance with other agents. In addition to classic inhibitory experiments, the propensity of drugs to induce ring-stage dormancy, characteristic of artemisinin resistance, must also be evaluated [109].

#### Single encounter radical cure and prophylaxis (SERCaP)

Proposed in malERA 2011, SERCaP remains a priority [5]. Radical cure means clearance of all asexual blood-stage forms, mature gametocytes, and *P*. *vivax*/*P*. *ovale* hypnozoites (Fig 1). Combination therapies of new chemical entities (NCEs) that are targeted to ‘single encounter, radical cure‘ are now in phase II clinical trials, with potential regulatory submission dates circa 2021 (S1 Table) [17].

The post-treatment prophylactic component of the SERCaP will come from the long half-life of the active pharmaceutical ingredients. Malaria parasite elimination will require new generations of single-encounter chemoprotection, to protect migrating populations and protect against epidemics in the later stages of elimination. These products would include molecules that provide chemoprotection by targeting the preerythrocytic stages (see TCP-4 specific attributes in [110]).

Reducing duration of dosing regimens, ideally to a single dose, increases adherence, be it for prevention or treatment [8]. Although better adherence improves effectiveness, it must be achieved without significantly increasing the risk of selection for drug-resistant parasites as a result of creating long periods of subtherapeutic drug levels. As a country or area approaches elimination, the remaining parasites are likely to be those most resistant to treatment, and drug classes with a low propensity to select for parasite resistance should be prioritised [111, 112]. The temptation to combine new drugs with old drugs with preexisting resistance, whilst simpler from a regulatory perspective, must be avoided to prevent novel agents being exposed as functional monotherapies when used against strains resistant to the older partner drug.

#### Severe malaria

Intravenous and intramuscular artesunate are currently the most effective and well-tolerated treatments for severe malaria [113,114], with rectal artesunate recommended for pre-referral treatment of children who cannot quickly access hospital care [106]. The potential spread of artemisinin resistance threatens the effectiveness of artesunate for treatment of severe malaria. Thus, new compounds with rapid activity against asexual blood stage parasites, suitable for parenteral administration, are needed for this critical indication (see TCP-1 specific attributes in [110]). The decline in the incidence of severe malaria in adults will require alternative development approaches, including the development of surrogate end points [115], as not enough patients will be available for large mortality studies [116]. However, sufficient safety data in adults would still be required for a phase III trial in African children with severe malaria.

#### Interrupting transmission

Drugs with activity against gametocytes in humans or that impair sporogony in the mosquito could help to interrupt transmission (Fig 1) [117]. While low-dose (0.25 mg/kg single dose) primaquine is currently recommended as a gametocytocidal following ACT treatment for *P*. *falciparum* malaria in areas of low transmission [16,118,119], NCE combination therapies with both therapeutic and transmission-blocking activity would simplify drug administration. High-throughput screening and clinical evaluation of compounds with transmission-blocking activity are now possible (Box 2) [59,120–125] and have yielded new leads, including more than a dozen from the Medicines for Malaria Venture toolbox with activity in the standard membrane feeding assay [126].

#### Global antimalarial drug development portfolio

There are at least 15 active projects in preclinical development or phase I or II clinical trials (S1 Table) [17]. A range of new chemotypes targeting new parasite pathways are available, with antimalarial drug development accelerated using CHMI models (Box 2). Two pairs of NCE combinations are in phase II clinical studies: the long-lasting synthetic endoperoxide artefenomel (OZ439) combined with the next-generation 4-aminoquinoline, ferroquine; and the imidazolopiperazine KAF156 combined with a new once-per-day lumefantrine formulation. This latter combination is also being explored as a 3-day regimen for use as a frontline agent in areas with ACT resistance. Single-dose effectiveness with an appropriate safety profile may require triple combination therapy. Notably, KAF156, DSM265, and MMV390048 have activity against *P*. *falciparum* liver stages and could be given as a single-dose treatment or once weekly for chemoprotection (S1 Table) [127]. TPPs and target candidate profiles with minimal essential and ideal attributes for single-encounter chemoprotection have been published [110].

### Antihypnozoite drugs

In areas of high transmission, such as Papua New Guinea, relapses cause approximately 4 of every 5 *P*. *vivax* infections [128]. Modelling suggests that for rapidly relapsing tropical *P*. *vivax* strains, effective relapse prevention has the potential to significantly reduce transmission [104]. In areas of seasonal transmission, relapses allow parasites to rapidly reestablish transmission once vector populations recover [129]. The 8-aminoquinoline primaquine is the only antirelapse therapy currently available (aside from chloroquine, to which there is extensive resistance), but treatment courses are 7–14 days, and poor adherence undermines effectiveness [130]. Tafenoquine is a candidate single-dose 8-aminoquinoline, showing high antirelapse efficacy in *P*. *vivax* infections when given with chloroquine [18]. Phase III clinical trials were completed in 2016, with regulatory submission anticipated in 2017. The impact of tafenoquine on transmission remains to be evaluated in post-registration CHMI and field trials. G6PD-deficient individuals cannot be given standard doses of 8-aminoquinolones; in addition, 8-aminoquinolones are considered contraindicated during pregnancy and lactation. As such, new antihypnozoite drug classes without these contraindications are needed. Discovery should be enhanced in the next 5 years through screening campaigns against *P*. *vivax* liver stages using stable human cell systems [131,132]. Additionally, humanised mouse models are facilitating drug development against this life cycle stage that thus far has been refractory to study [133].

### Seasonal malaria chemoprevention (SMC)

In areas where malaria is seasonal, providing SMC by monthly treatment with long-lasting antimalarial drugs greatly reduces malaria burden in children under 5 years of age [134,135]. Modelling studies indicate the potential for reducing transmission to very low levels if SMC is combined with long-lasting insecticidal nets (LLINs) at 80% coverage and expanded to children up to 10 years of age [136]. Sulfadoxine-pyrimethamine + amodiaquine (SPAQ) is used for SMC in the Sahel; drug resistance prevents SPAQ use in eastern and southern Africa, and there are concerns that resistance may spread to the Sahel. Thus, alternative drugs are required, ideally with simplified dosing regimens.

### Endectocides

Endectocides are an alternative approach to malaria control whereby humans and/or livestock are given agents with insecticidal activity, resulting in reduced survival of the vector upon blood feeding and impairment of malaria parasite transmission [137]. Modelling studies suggest that the endectocide ivermectin could help achieve transmission interruption as an additional intervention in settings where mass treatment strategies with ACTs alone would be insufficient to accomplish elimination [138]. A research agenda was proposed in 2013 outlining the path for ivermectin use in malaria [139], with a number of studies in different settings underway. A WHO expert group recently examined this concept, with findings anticipated in 2017. The antimosquito properties of veterinary and other candidate endectocides are also being explored.

### Novel formulations

An interesting possibility is the application of nanomilling and related technologies to develop long-acting drug formulations, which are being investigated for long-term HIV preexposure prophylaxis and in combination with contraception in so-called multipurpose prevention technologies (MPTs) [140–143]. Such long-acting drug formulations could potentially allow chemoprotection over several months from a single injection. Application to new generations of transmission-blocking molecules or endectocides could provide tools that reduce or prevent transmission over an entire transmission season.

### Challenges

Attrition rates in antimalarial drug development are comparable with those in other infectious diseases [126]. Thus, discovery momentum needs to be maintained at high levels if new drugs are to reach licensure. A major challenge in registering NCEs for malaria is assembling the substantial clinical safety data required for regulatory approval, particularly in the key target populations of infants and pregnant women. Thus, reproductive safety should be evaluated early in preclinical development to prioritise investment in NCEs with appropriate preclinical profiles.

With the introduction of NCEs during the next 5 years, pharmacovigilance needs strengthening in malaria-endemic areas. This is also a prerequisite for safe deployment of current ACTs and next-generation treatments during mass treatment programmes targeted at populations that include individuals with subclinical malaria or who are infection free.

In the next 5–10 years, there is a need to enrich the early-stage portfolio with new antihypnozoite drugs beyond the current 8-aminoquinolones. Cell biology and the animal models supporting drug discovery for new antihypnozoite agents have progressed significantly but are still not amenable to high-throughput screening programmes [144,145]. Clinical trials for relapse prevention take 6–12 months, much longer than treatment trials. Additionally, relapses can be caused by hypnozoites that are homologous or heterologous to the initial infection and cannot, therefore, be distinguished from recrudescence or reinfection [146–148], except by the physical removal of treated patients from transmission areas, e.g., repatriated soldiers and travellers.

Although NCEs active against artemisinin-resistant isolates are in development, better strategies are needed to deploy drugs to delay or prevent the emergence of drug resistance, such as measures to tackle counterfeiting or manufacturing of poor-quality medicines, drug sequencing, multiple firstline therapies, and exploiting competing resistance mechanisms, as discussed elsewhere in the malERA Refresh series [8].

## Vaccine research agenda

The Malaria Vaccines Technology Roadmap was updated in 2013 [34], with the goal of developing by 2030 vaccines for *P*. *falciparum* and *P*. *vivax* that have a protective efficacy of at least 75% against clinical malaria and/or reduce transmission of the parasite. The roadmap outlines key priorities in research, vaccine development, key capacities, policy, and commercialisation. The research issues in malaria vaccines are discussed below, but key to their success will be ensuring an efficient and cost-effective distribution system and redirection of the health system from delivering malaria treatment to prevention and transmission interruption [9].

### Vaccines to prevent clinical malaria and interrupt transmission

A preerythrocytic vaccine to interrupt malaria transmission (PE-VIMT) that completely prevents liver-stage infection for a significant duration (e.g., at least 1 transmission season) would prevent parasitaemia and gametocyte generation and therefore interrupt onward transmission (Fig 1). Although RTS,S is a preerythrocytic vaccine, demonstrating modest efficacy in preventing clinical malaria, prevention of infection and transmission were not evaluated in the late-stage clinical trials (Box 1). More recently, a delayed fractional dose regimen of RTS,S with improved efficacy against a parasite transmission (mosquito-to-human) end point may be considered for transmission-blocking potential (Box 1) [31]. Several next-generation preerythrocytic candidates are in clinical development, including multistage (including asexual blood-stage and/or sexual/sporogonic/mosquito-stage targets) combinations and prime-boost strategies, as well as irradiated or genetically attenuated sporozoites (S2 Table) [35,149]. Future directions need to ensure a widely acceptable route of administration, optimised dose regimens, and lower inoculum sizes.

Blood-stage vaccines are an alternative and complementary approach to PE-VIMT. Blood-stage vaccines that interrupt malaria parasite transmission (BS-VIMTs) by efficiently clearing blood-stage infections would limit gametocyte densities and the duration that a person is infectious, thus reducing human-to-mosquito malaria parasite transmission (Fig 1). Several promising *P*. *falciparum* vaccine candidates are in clinical development [150], including the unstructured peptide P27A, the well-studied PfRH5, and the 2 placental malaria vaccine candidates PAMVAC and PRIMVAC (S2 Table). Innovative new concepts in next-generation malaria vaccine protein subunit design are being explored to develop highly effective multicomponent/multistage/multiantigen formulations [151].

### Vaccines that only interrupt malaria parasite transmission

Sexual-sporogonic-mosquito-stage vaccines to interrupt transmission (SSM-VIMT) inhibit parasite transmission from human to mosquito, through reducing gametocytes’ ability to infect mosquitoes or by interfering with parasite development (sporogony) within the mosquito (Fig 1). As the potential benefit to the recipient is both delayed and indirect, the PATH Malaria Vaccine Initiative and partners are exploring potential regulatory and policy approaches with the United States Food and Drug Administration and WHO, respectively [152,153]. Progress has been made, with a design proposed for a phase III study [154]. The Pfs25 antigen is expressed on the surface of zygotes and ookinetes in the mosquito midgut, and various attempts to improve immunogenicity and transmission-blocking activity have been undertaken (S2 Table) [36,153]. The most clinically advanced is Pfs25-EPA (a detoxified form of exotoxin A from *Pseudomonas aeruginosa*) conjugate [155]. Most recently, Pfs25 has been fused with IMX313, a molecular adjuvant, and expressed in chimpanzee adenovirus 63 (ChAd63) and Modified Vaccinia Virus Ankara (MVA) viral vectors and as a secreted protein nanoparticle [156]. The research agenda has broadened to include other SSM-VIMT antigens, including Pfs230 and Pfs48/45 (S2 Table).

### Vaccines for *P*. *vivax/P*. *ovale*

A vaccine that could prevent *P*. *vivax/P*. *ovale* infection and hypnozoite formation, target hypnozoites, or prevent disease, thereby interrupting transmission and draining the hypnozoite reservoir, would be a significant step for accelerating malaria elimination (Fig 1). *P*. *vivax* is now included in the Malaria Vaccine Technology Roadmap strategic goals [34]. While basic research in *P*. *vivax* has increased in recent years, no vaccine candidate has progressed past early human studies (S3 Table) [35,36]. Three preerythrocytic vaccines have reached clinical trials [157–159]. A blood-stage vaccine targeting the Duffy-binding protein region II has progressed to early clinical trials [160], though combination with other blood-stage antigens is likely necessary to achieve high growth inhibition. The Pvs25 antigen is also being investigated as an SSM-VIMT. The recent development of *P*. *vivax* CHMI systems allows evaluation of vaccine efficacy [157,161]. Also, publication of the *P*. *ovale* and *P*. *malariae* genomes facilitates antigen discovery for these parasites [162].

### Adjuvants, delivery platforms, and desired human immune responses

Most (but not all) malaria vaccines in development are based on *Plasmodium* protein subunits and have shown limited immunogenicity in humans. Suitable adjuvants and delivery platforms are therefore needed to elicit the desired immune response and induce significant protection from infection and disease without unacceptable collateral inflammation [163]. There are few adjuvants licensed for human use and there is a need to (1) better define the desired human immune response; (2) facilitate access to adjuvants in development and ensure downstream availability, affordability, and acceptability; (3) develop more specific targeted adjuvants that boost desired immune responses while maintaining acceptable safety; and (4) match individual adjuvants to individual vaccine candidates depending on the postulated mechanism of action while maintaining compatibility for combination vaccines.

### Prophylactic biologics

Monoclonal antibodies are another potential tool. Recently, the major barriers of cost are being overcome through improvements in manufacturing and high-expressing cell lines [164]. A recent report estimated that the cost of goods for monoclonal antibodies had reduced 10-fold, from thousands of dollars per gram to around $100 per gram, with the costs of developing these agents comparable to other therapeutic drugs and vaccines [165]. Additionally, the volume and frequency of administration of monoclonal antibodies have been reduced by improvements in potency and pharmacokinetics [166]. There has been a significant increase in the number of validated vaccine targets, and now monoclonal antibodies can be studied early in clinical development for their ability to provide immediate protection in CHMI models, either singly or in combination [167]. Antibodies are less prone to the off-target safety and toxicity issues that often plague small molecule development and thus offer significant advantages for deployment in vulnerable populations, including the immunocompromised and pregnant women. In the context of elimination, monoclonal antibodies with suitable pharmacokinetics/pharmacodynamics could represent an alternative to active immunisation by VIMTs or transmission-blocking drugs. As with any tool, prophylactic biologicals will need to be designed to meet the needs and capabilities in target settings.

### Challenges

To achieve malaria elimination, vaccines would ideally be able to prevent infection by all 5 species of human malaria parasites. While humans are the major (if not only) reservoir for 4 of 5 *Plasmodium* ssp., zoonotic *P*. *knowlesi* presents a unique challenge for elimination given continuous sylvatic transmission [168]. If a ‘*Plasmodium*’ vaccine targeting all human-infecting species is not feasible, then vaccines are required against individual species. It remains to be determined whether experience gained in the development of *P*. *falciparum* vaccines can, in fact, inform approaches to other malaria species or whether new strategies are required.

Similar to drugs used in MDA, vaccines for mass inoculation need to be safe for use in pregnant women and children. Demonstrating safety across the target population is particularly important for vaccines that only prevent transmission and have an indirect benefit to the recipient.

For malaria vaccine candidates, there is limited information on immune correlates that may predict efficacy in the chosen indication. Antigenic diversity of many of the malaria vaccine targets [169,170] adds additional complexity to predicting efficacy and enables parasites to evade host immune responses, potentially leading to vaccine escape mutants [171,172].

There is also incomplete understanding of the development and maintenance of either naturally acquired or vaccine-induced human immunity to *Plasmodium*. A predictable ‘age shift’ in peak incidence of malaria associated with vaccines with modest and/or waning efficacy in children who have not acquired full natural immunity must be anticipated and appropriately managed [173]. The challenge of maintaining individual and population-based (herd) immunity may increase as circulating parasite prevalence declines during the later stages of elimination. Thus, rationally designing vaccines that induce long-lasting immunity in semi-immune adults and provide broad cross strain protection presents formidable challenges.

Finally, as with drugs, parasite genetic diversity and rich population structures, particularly in high-transmission settings, indicate the potential for differential parasite-specific efficacy and selection of resistant *Plasmodium*. The former has been observed in vaccine field studies, including a recent genetic analysis associated with a large phase III trial of RTS,S/AS01 [170]. However, there are no data regarding whether implementing malaria vaccination induces parasite resistance in the whole population of infected individuals.

## Vector control research agenda

### Insecticide-based interventions

LLINs are currently the single most important malaria control intervention, responsible for approximately 68% of malaria cases averted in Africa [174]. However, emerging resistance to insecticides among *Anopheles* mosquitoes threatens to reverse these gains [175,176]. New insecticides with different modes of action are urgently needed to deter resistance development. In response, ‘Innovation to Impact’ was initiated in 2013 with an aim to transform the process for developing and delivering life-saving vector control products for diseases caused by vector-borne pathogens. More than 30 different stakeholder groups are involved, including industry, global evaluation and regulatory bodies, procurers, local and national representatives, and donors [42,43].

Twelve insecticide products are currently available for vector control, confined to 4 chemical classes (pyrethroids, organochlorines, organophosphates, and carbamates), although only pyrethroids are widely used for LLINs. Several combination LLINs consisting of different insecticide classes or incorporating the synergist piperonyl butoxide are in late-stage development (Table 1) [39–41,177–181]. Similar to LLINs, long-lasting insecticide-treated hammocks could be effective in remote areas; however, the lifespan of these interventions has a significant impact on cost-effectiveness, and exploration of technologies to increase durability is needed [182].

**Table 1:** Insecticides for indoor residual spraying (IRS) under World Health Organization Pesticide Evaluation Scheme (WHOPES) evaluation and long-lasting insecticidal nets (LLINs) in late-stage development [39–41,177,178]*.

Application	Product	Insecticide(s)
IRS	Phantom	Chlorfenapyr (phase III)
	SumiShield	Clothianidin (phase II)
	Fludora Fusion	Deltamethrin + clothianidin (phase II)
LLINs	DawaPlus 2.0	Deltamethrin coated on polyester
	LifeNet	Deltamethrin incorporated into polypropylene
	MiraNet	Alpha-cypermethrin incorporated into polyethylene
	Panda Net 2.0	Deltamethrin incorporated into polyethylene
	Yahe	Deltamethrin coated on polyester
LLINs + PBO	Olyset Plus	Permethrin + PBO incorporated into polyethylene
	PermaNet 3.0	Deltamethrin coated on polyester side panels; deltamethrin + PBO incorporated into polyethylene (roof)
	Veeralin	Alpha-cypermethrin and PBO incorporated into polyethylene
Combination LLINs	Olyset Duo	Pyriproxyfen and permethrin incorporated into polyethylene
	Interceptor G2	Alpha-cypermethrin + chlorfenapyr coated on polyester

*March/April 2016.

PBO, piperonyl butoxide

After screening around 4 million compounds, 3 new insecticides have progressed to development, with registration typically taking 5–7 years [44,178]. These new insecticides are primarily pyrethroid alternatives for use in LLINs but also would be expected to have use in IRS. For IRS, 2 long-lasting formulations of existing compounds have become available: a microencapsulated formulation of the organophosphate insecticide pirimiphos methyl in 2012 [37] and a polymer-enhanced suspension of deltamethrin in 2013 [38]. The Next Generation IRS project is a market intervention to accelerate uptake and increase use of long-lasting IRS products [183]. Additional long-lasting insecticides suitable for IRS are in development (Table 1).

New ways of using insecticides require more extensive field evaluation, e.g., technological advances for improving spraying techniques [184], timing of insecticide deployment to coincide with seasonal transmission, slow-release polymer-based wall linings [185,186], insecticide-treated eave tubes or eave ‘bricks’ combined with house screening, and electrostatic coatings to enhance insecticide bioavailability [187].

### Vector behaviour and outdoor targeting

Greater understanding of vector behaviour is needed, including the behavioural adaptations of vectors in response to control measures, such as changes in biting times, resting locations, and rates of zoophagy [188–194]. Improved targeting of specific vector behaviours—particularly sugar feeding, oviposition, mating, dry-season survival, and swarming behaviour—and zooprophylaxis are generating novel approaches to vector control, with potential application across transmission settings [195,196].

Long-standing evidence that malaria parasite transmission to humans occurs outdoors in Southeast Asia and South America and increasing evidence of outdoor transmission in sub-Saharan Africa [3,197–202] suggest a specific need for interventions that target mosquitoes outside dwellings. Attractants/traps are a potential new area of mosquito control that can be applied both indoors and outdoors. These include attractive toxic sugar baits [203,204] and sound traps, which lure male mosquitoes by broadcasting sounds similar to the wingbeats of female mosquitoes [195,203]. All major malaria parasite vectors in Africa mate in swarms [206], which are easily found and recognised, appear to be stable over time, and exist in a defined space [195]. This facilitates close targeting either with insecticides or traps [195]. Spatial repellents are another approach, releasing into the air volatile chemicals that prevent human–vector contact within the treated space (indoor or outdoor). Guidelines for efficacy testing are now available [205,207], and evaluation in outdoor settings is needed [208,209].

### Environmental management and larval source management

Environmental management, such as improved housing and water management, can be highly effective in specific epidemiological and environmental settings [210]. The best of these environmental management approaches require further investigation in tropical climates and resource-poor settings to establish their epidemiological impact in these settings [210,211]. Mosquito larval source management is the management of water bodies that are potential larval habitats to prevent immature mosquitoes developing into adults, either by environmental management or application of larvicides [45]. Larval source management has been highly effective in certain situations [211], but as this is a resource-intensive activity, better definition of the appropriate requirements and approaches across a wider range of settings is needed.

### Genetic approaches

There are 2 main strategies for genetically modifying mosquito populations: (1) population suppression, whereby mosquitoes are modified in such a way that upon mating with the wild type the resulting progeny are either sterile or dysfunctional, and (2) population alteration or replacement, in which the mosquitoes are modified in such a way that upon mating with the wild type, the resulting progeny are rendered refractory to malaria parasite infection. Genetic approaches now appear operationally feasible given recent advances in molecular biology, such as the efficient genome-editing techniques based on CRISPR/Cas9 and other approaches [10,212,213].

The sterile insect technique was the first attempt at genetic population suppression, whereby large numbers of irradiated sterile males are released with the hope that females mate unsuccessfully [214]. A more recent development is the release of insects carrying a dominant lethality, with the progeny of females mating with genetically modified males inheriting a lethal gene [215,216].

Gene drive systems exploit ‘homing’ endonucleases. These induce the lateral transfer of an intervening DNA sequence to a homologous allele that lacks that sequence, thereby changing a heterozygote into a homozygote. Conventional homing endonucleases have been reengineered to recognise mosquito genes [217] and can rapidly increase the frequency of desirable traits in a mosquito population [218]. Technical feasibility has been demonstrated for a CRISPR/Cas9-based gene drive system with the potential to reduce mosquito populations [219] or make them less able to transmit malaria parasites [220].

There is also the potential for symbiont-mediated biocontrol in malaria *Anopheles* mosquito populations, as suggested by recent successes achieved against *Aedes aegypti* (e.g., *Wolbachia*-mediated pathogen interference for dengue control). A further step is paratransgenesis, whereby a vector symbiont (virus, bacteria, or fungi) is engineered to express ‘effector’ molecules within the vector that are deleterious to the pathogen. Genetic modification of symbionts is easier than it is for mosquitoes and is independent of mosquito species, providing the symbiont can survive and colonise the host [221], and laboratory studies have shown promise [222].

There are environmental uncertainties associated with widespread distribution of technologies involving genetic manipulation of pathogens, vectors, or their symbionts [10,212]. Phased testing starting at a small scale is recommended, though the parameters for ecological risk assessment are not well understood.

### Challenges

The development of new insecticides will need to outpace the expansion of insecticide-resistant alleles in mosquito populations, and new products will need to be deployed to effectively combat behavioural resistance [8]. The imperfect correlation between entomological indicators and disease incidence complicates the accurate assessment of new vector control tools. Randomised controlled trials are expensive and time consuming, and new pathways should be explored for generating evidence for large-scale implementation of new interventions. Increasing fine-scale heterogeneity, in human and vector subpopulations and in geographic space, means that no single set of interventions will be effective across large areas or districts. Notwithstanding resource availability, the challenge is to understand which combinations of vector control measures are appropriate in different settings and how their effects can be augmented with other interventions (e.g., endectocides, transmission-blocking drugs, and vaccines) [7]. Targeting mosquito dormancy remains a challenge in large part because of the paucity of mechanistic evidence by which vectors persist during the dry-season (e.g., diapause [aestivation] and long-distance migration) [223]. Finally, it is important to note that there are very few trained entomologists in national malaria control programs, especially at the district level. To develop and implement vector-targeted interventions, greater entomology capacity building is required.

## Conclusions

There are overarching areas in which greater knowledge is required to understand the utility of current interventions and define which products and strategies might be required going forward. Novel tools may allow further investigation of knowledge gaps, and some may be bridgeable (Table 2). The R&D agenda for tools for elimination is summarised in Box 3.

**Table 2:** Knowledge gaps and tools to potentially bridge the gaps.

Knowledge gaps	Tools to potentially bridge the gaps
High-to-low transmission	
• Why do transmission rates remain high even when case management and vector control have high coverage?	• Methods that are protective against infection and interrupt transmission (Fig 1).
• At which point should interventions specifically directed at reducing transmission be introduced?	• Robust mathematical and laboratory models of transmission and impact of combination interventions.
• What is the contribution of the subclinical reservoir to transmission in high-transmission settings?	• Sensitive point-of-care tests to detect transmission reservoirs and enable evaluation of interventions.
• In vector control, which factors drive changes in transmitting species?	• Prevention or control measures effective against all species.
Low-to-zero transmission	
• How best can the remaining sources of transmission be identified?	• Affordable, rapid, sensitive screening techniques to identify populations generating infectious gametocytes.
• What is the impact of nonhuman malaria parasite transmission on the effectiveness of vector control?	• Vector control measures with efficacy independent of nonhuman transmission.
• How can transmission be measured when it is low or zero?	• See Malaria Eradication Research Agenda (malERA) Refresh ‘Characterising the reservoir and measuring transmission’ [73].
• How can approaches to false-positive diagnostic tests be addressed?	• Development of highly sensitive and specific tests, along with combination testing algorithm/protocols to identify false positives.
• How can the *P*. *vivax*/*P*. *ovale* hypnozoite reservoir be identified and targeted?	• Development of hypnozoite diagnostics, and/or antihypnozoite drugs/vaccines that are safe enough for use in population-based administration.
• How can heterogeneity in transmission be managed?	• Interventions that are safe and cost-effective enough to be used across wider populations.
Maintaining zero transmission	
• How can the efficacy of tools be measured when transmission is zero?	• Development of validated surrogate end points of efficacy.
• What are the drivers of epidemic malaria?	• Modification of vector populations to decrease epidemic potential; tools for epidemic response, including for nonimmune populations.

**Box 3. Research and development agenda for tools for elimination**

**Diagnostics**

*Detecting transmission* *potential*

- Malaria diagnostic tools best suited for detection of low-density, subclinical infections
- Assay for detecting infectious gametocytes
- *P*. *vivax/P*. *ovale* hypnozoite detection methods
- Noninvasive diagnostic tests

*Directing* *treatment*

- Stable, valid, specific, and sensitive rapid diagnostic tests (RDTs) that do not depend on histidine-rich protein 2 (HRP2)
- Detection of drug-resistant parasites
- RDTs that detect and differentiate all relevant human *Plasmodium* ssp. pathogens
- Multiplexed point-of-care tests for acute febrile illness

*Special* *populations*

- Affordable, simple, and accurate point-of-care tests for glucose-6-phosphate dehydrogenase (G6PD)-deficient individuals and pregnant women

**Drugs**

*Drugs* *for prevention and treatment*

- Drugs that overcome resistance to existing drugs, particularly artemisinin resistance
- A suite of combination drugs with different or competing resistance profiles
- New drugs for prophylaxis
- Simplifying therapy, with the potential objective of a single encounter radical cure and prophylaxis (SERCaP)
- New regimens for use in seasonal malaria chemoprevention (SMC) outside the Sahel and to potentially replace sulfadoxine-pyrimethamine + amodiaquine (SPAQ)

*Drugs* *to interrupt transmission*

- Investigation of the impact of low-dose primaquine in different settings
- New drugs with transmission-blocking potential
- Drug combinations incorporating both asexual and transmission-blocking activity
- Evaluate the impact of transmission-blocking drugs on pathogen resistance development and investigate optimal deployment strategies
- Endectocides for use in humans and animals

*Antihypnozoite* *drugs*

- Evaluation of *P*. *vivax* transmission reduction potential with tafenoquine via relapse prevention (draining of hypnozoite reservoir)

*Special* *populations*

- New small molecules or antibodies with a potential indication for use during pregnancy

**Vaccines**

*RTS*,*S*

- Further evaluation of RTS,S to determine the potential for increased efficacy with alternative dosing regimens
- Assessment of RTS,S in combination with other interventions (e.g., SMC) and in other epidemiological settings and populations

*New* *vaccines*

- Defining the required attributes of preerythrocytic or blood-stage vaccines to achieve transmission-blocking activity
- New preerythrocytic and or blood-stage vaccines, ideally with transmission-blocking potential
- A first sexual-sporogonic-mosquito-stage vaccine to interrupt transmission (SSM-VIMT)

*Vaccines against* P. vivax/P. ovale

- Vaccines that prevent infection and hypnozoite formation, target hypnozoites, or can interrupt transmission to eventually eliminate the hypnozoite reservoir

Adjuvants

- Access to a broader choice of adjuvants with improved risk–benefit profiles

*Prophylactic* *biologics*

- Development of monoclonal antibodies (mAbs) and combinations of recombinant multi-mAbs products

**Vector** **control**

*Insecticides and long-lasting insecticidal nets* *(LLINs)*

- New insecticides and combinations of insecticides to overcome vector resistance
- Nonpyrethroid insecticides for LLINs
- Investigation of new insecticide deployment strategies
- LLINs with improved durability

*Environmental* *management*

- Formal investigation of larval source management in a greater variety of settings
- Development of long-lasting safe larvicides
- Development of cost-effective and socially acceptable environmental management interventions

*Genetic* *approaches*

- Development of scalable genetic approaches
- Development of environmentally and socially responsible methods for field testing transgenic organisms

*Exploiting* *vector behaviour*

- Novel interventions to target populations and behaviours
- Increased entomological support for key decisions by national malaria programmes

*Combination* *and mapping*

- Modelling to suggest the most effective and efficient combinations of vector control for different settings
- Developing operationally relevant mapping tools to identify and target residual transmission

**‘Tools to develop** **tools’**

- Validating outcomes from animal and human infection models that predict a reduction in transmission in real-life settings
- Robust mathematical and laboratory models of transmission and impact of combination interventions
- Increased understanding of parasite–host immunity and mechanisms of acquired and vaccine-induced protective and transmission-blocking immunity
- Development of high-throughput screening assays and evaluation assays for the identification and selection of compounds with neglected profiles (e.g., antihypnozoite activity)

Transmission can remain high even with high coverage of good-quality case management and vector control. Thus, products and strategies directed specifically at accelerating elimination by targeting transmission are needed. Interventions may only achieve transmission reduction when deployed in certain populations or settings. Conversely, some populations and settings may require specific measures for transmission reduction, for example, pregnant women and infants, migrant workers, subclinical parasitaemia, or addressing outdoor transmission. The availability of new interventions is expanding, but developing algorithms for their rational combination and deployment in packages to decrease transmission is a key research need that requires modelling support [7]. Cost-effectiveness is an important determinant of whether particular interventions are adopted in public health programmes [9].

New products and strategies are needed to overcome parasite drug resistance and vector resistance to insecticides [8]. Prevalence of vaccine escape mutants has been highlighted as a potential issue if vaccines become widely used [170–172]. Thus, product development must continue, and strategies for phased replacement are needed as effectiveness wanes. New product discovery and development requires investment in basic science [10], the alignment of regulatory structures to expedite product registration, and continued investment in pharmacovigilance and surveillance. Funding organisations and malaria programmes also need to be convinced that tools are impactful and cost-effective [9]. However, measuring the efficacy of tools that potentially impact transmission is problematic, particularly at the extremes of transmission [154]. Thus, new diagnostics and screening methods are required to assess tool efficacy in low-transmission settings and determine their contribution to maintaining zero transmission [73]. Moreover, the development of new diagnostics with improved sensitivity, or for specific tasks such as resistance surveillance, may fundamentally change our perception of malaria parasite transmission and our understanding of the most appropriate interventions required to interrupt transmission.

Finally, developing new tools can be expensive. When the malaria burden is significant, the economic case for innovation is clear. However, as the malaria burden decreases, the economic argument for continued development becomes more nuanced. Public–private partnerships, which first emerged 15 years ago, have demonstrated the ability to partner and drive development for a variety of tools, including diagnostics (e.g., PATH and Foundation for Innovative New Diagnostics), drugs (e.g., Medicine for Malaria Venture and formerly Drugs for Neglected Diseases Initiative), vaccines (e.g., PATH Malaria Vaccine Initiative and European Vaccine initiative), and vectors (e.g., Innovative Vector Control Consortium and more broadly Malaria No More). New business models to attract and engage industry in developing tools for the elimination should be considered as well. Interventions will be directed at increasingly smaller populations, but these populations often represent the most difficult contexts in which to achieve elimination, and multiple interventions may be required. Once a country achieves elimination, there is the temptation to scale back infrastructure and interventions for malaria. This risks triggering a potentially lethal outbreak that could be difficult to reeliminate or even contain. There are numerous examples from earlier malaria elimination campaigns in the 1950s and 1960s of initial successes that were followed by resurgence as campaigns were deprioritized or discontinued administratively, financially, and technically. Unless malaria can be completely eradicated, interventions to maintain malaria elimination and a reserve of effective measures to counter malaria outbreaks will always be needed. However, if the right products and strategies are developed, and if they are used efficiently, effectively, and consistently, malaria eradication is an achievable goal.

## Supporting information

S1 Table Antimalarial drugs in preclinical and early clinical development. (PDF)

S2 Table Vaccines against *P*. *falciparum* in clinical development. (PDF)

S3 Table Vaccines against *P*. *vivax* in clinical development. (PDF)

S1 Text Summary of progress in malaria diagnostics since the initial Malaria Eradication Research Agenda (malERA) initiative and remaining gaps. (DOCX)

S2 Text Summary of progress in malaria drugs since the initial Malaria Eradication Research Agenda (malERA) initiative and remaining gaps. (DOCX)

S3 Text Summary of progress in malaria vaccines since the initial Malaria Eradication Research Agenda (malERA) initiative and remaining gaps. (DOCX)

S4 Text Summary of progress in malaria vector control since the initial Malaria Eradication Research Agenda (malERA) initiative and remaining gaps. (DOCX)

## Abbreviations

ACT: artemisinin-based combination therapy
BS-VIMT: blood-stage vaccine that interrupts malaria parasite transmission
ChAd63: chimpanzee adenovirus 63
CHMI: controlled human malaria infection
G6PD: glucose-6-phosphate dehydrogenase
iiPCR: insulated isothermal polymerase chain reaction
IRS: indoor residual spraying
LAMP: loop-mediated isothermal amplification
LDH: lactate dehydrogenase
LDR-FM: ligase detection reaction fluorescent microsphere
LLIN: long-lasting insecticidal net
malERA: Malaria Eradication Research Agenda
MDA: mass drug administration
MPT: multipurpose prevention technology
MVA: Modified Vaccinia Virus Ankara
NCE: new chemical entity
NINA-LAMP: noninstrumented nucleic acid amplification LAMP
PCR: polymerase chain reaction
PE-VIMT: preerythrocytic vaccine to interrupt malaria transmission
PfHRP2: *Plasmodium falciparum* histidine-rich protein 2
QT-NASBA: quantitative nucleic acid sequence-based amplification
qPCR: quantitative PCR
R&D: research and development
RDT: rapid diagnostic test
SERCaP: single encounter radical cure and prophylaxis
SMC: seasonal malaria chemoprevention
SNP: single nucleotide polymorphism
SPAQ: sulfadoxine-pyrimethamine + amodiaquine
SSM-VIMT: sexual-sporogonic-mosquito-stage vaccines to interrupt transmission
TPP: target product profile
WHO: World Health Organization
