# Supplementary material for: malERA: An updated research agenda for diagnostics, drugs, vaccines, and vector control in malaria elimination and eradication
Source: PLoS Med. 2017 Nov 30;14(11):e1002455. doi: 10.1371/journal.pmed.1002455 (PMC5708606; doi:10.1371/journal.pmed.1002455)
Supplement: S1 Table — (PDF) [file pmed.1002455.s001.pdf]

PHASE	DRUG	TARGET CANDIDATE PROFILES		
PRECLINICAL	DDD498	Asexual blood-stage activity	Transmission reduction	Chemoprevention
PRECLINICAL	PA92	Asexual blood-stage activity	Transmission reduction	
PRECLINICAL	MMV253	Asexual blood-stage activity		
PRECLINICAL	GSK030	Asexual blood-stage activity	Transmission reduction	
PRECLINICAL	DSM421	Asexual blood-stage activity		Chemoprevention
PRECLINICAL	UCT943	Asexual blood-stage activity		
PRECLINICAL	AN762	Asexual blood-stage activity	Transmission reduction	Chemoprevention
HUMAN VOLUNTEERS	MMV048	Asexual blood-stage activity	Transmission reduction	Chemoprevention
HUMAN VOLUNTEERS	P218	Asexual blood-stage activity		Chemoprevention
HUMAN VOLUNTEERS	SJ733	Asexual blood-stage activity	Transmission reduction	
HUMAN VOLUNTEERS	DSM265	Asexual blood-stage activity		Chemoprevention
PATIENT EXPLORATORY	Artefenomel (OZ439)	Asexual blood-stage activity	Transmission reduction	
PATIENT EXPLORATORY	Cipargamin (KAE609)	Asexual blood-stage activity	Transmission reduction	
PATIENT EXPLORATORY	KAF156-Lumefantrine	Asexual blood-stage activity	Transmission reduction	Chemoprevention
PATIENT EXPLORATORY	DSM265	Asexual blood-stage activity	Chemoprevention	

- 🔵 Asexual blood-stage activity
- 🧍 Transmission reduction
- 🛡️ Chemoprevention
