# Supplementary material for: malERA: An updated research agenda for diagnostics, drugs, vaccines, and vector control in malaria elimination and eradication
Source: PLoS Med. 2017 Nov 30;14(11):e1002455. doi: 10.1371/journal.pmed.1002455 (PMC5708606; doi:10.1371/journal.pmed.1002455)
Supplement: S2 Table — (PDF) [file pmed.1002455.s002.pdf]

PHASE	VACCINE	ANTIGEN	ACTION
PHASE VI	RTS,S/AS01$_E$	Circumsporozoite protein	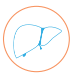
PHASE II	RTS,S/AS01$_E$ (fractional dose)	Circumsporozoite protein	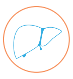
	ChAd63/MVA ME-TRAP + RTS,S	Thrombospondin-related adhesive protein + multiple epitopes circumsporozoite protein	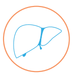
	PfSPZ Vaccine	Whole, irradiated *P. falciparum* sporozoites	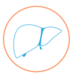
	GMZ2	glutamate-rich protein, merozoite surface protein 3	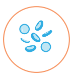
	MSP3 [181-276]	Merozoite surface protein 3	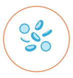
	NMRC-M3V-Ad-PfCA	Circumsporozoite protein and apical membrane antigen 1	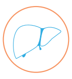 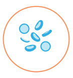
	PfPEBS	Lyophilised PEBS Synthetic Protein	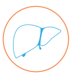 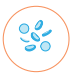
PHASE I	Pfs25 VLP	Zygote 25kDa surface protein	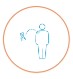
	ChAd63/MVA ME-TRAP + Matrix M™	Thrombospondin-related adhesive protein + multiple epitopes	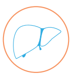
	PfCelTOS FMP012	Cell-traversal protein for ookinetes and sporozoites	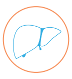
	CSVAC	Circumsporozoite protein	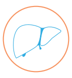
	R21/AS01B	Circumsporozoite protein	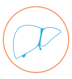
	R21/Matrix-M1	Circumsporozoite protein	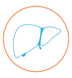
	PfSPZ-CVac	Whole, infectious *P. falciparum* sporozoites chemo-attenuated *in vivo*	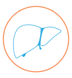
	PfAMA1-DiCo	Apical membrane antigen 1	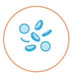
	P27A	Malaria protein PFF0165c fragment	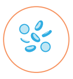
	BK-SE36	N-terminal domain of serine repeat antigen	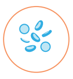
	ChAd63 RH5 +/- MVA RH5	Malaria invasion ligand RH5	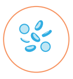
	PAMVAC	VAR2CSA fragment	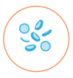
	PRIMVAC	VAR2CSA fragment	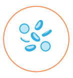
	Pfs25-EPA/Alhydrogel	Zygote 25kDa surface protein	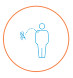
	Pfs230D1M-EPA/Alhydrogel and/or Pfs25-EPA/Alhydrogel	Zygote 25kDa surface protein , gamete 230kDa surface protein D1M	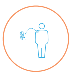
	ChAd63/MVA Pfs25-IMX313	Zygote 25kDa surface protein	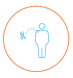

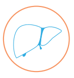 Pre-erythrocytic (liver-stage) vaccine

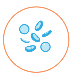 Blood-stage vaccine

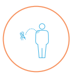 Vaccines that target sexual, sporogonic, and/or mosquito-stage antigens to interrupt malaria parasite transmission (SSM-VIMT)
