# Supplementary material for: malERA: An updated research agenda for diagnostics, drugs, vaccines, and vector control in malaria elimination and eradication
Source: PLoS Med. 2017 Nov 30;14(11):e1002455. doi: 10.1371/journal.pmed.1002455 (PMC5708606; doi:10.1371/journal.pmed.1002455)
Supplement: S3 Table — (PDF) [file pmed.1002455.s003.pdf]

PHASE	VACCINE	ANTIGEN	ACTION
PHASE I/IIa	**VMP001/AS01$_B$**	Circumsporozoite protein	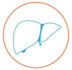
PHASE I	**PvCSP-LSP**	Circumsporozoite protein	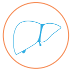
	**ChAd63-MVA-PvTRAP**	Thrombospondin-related adhesive protein	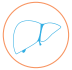
	**ChAd63/MVA PvDBP**	Duffy-binding protein region II	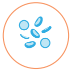
	**Pvs25H**	Zygote 25kDa surface protein	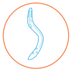

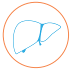 Pre-erythrocytic (liver-stage) vaccine

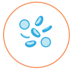 Blood-stage vaccine

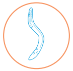 Vaccines that target sexual, sporogonic, and/or mosquito-stage antigens to interrupt malaria parasite transmission (SSM-VIMT)
