# Supplementary material for: malERA: An updated research agenda for diagnostics, drugs, vaccines, and vector control in malaria elimination and eradication
Source: PLoS Med. 2017 Nov 30;14(11):e1002455. doi: 10.1371/journal.pmed.1002455 (PMC5708606; doi:10.1371/journal.pmed.1002455)
Supplement: S1 Text — (DOCX) [file pmed.1002455.s004.docx]

**Diagnostics**

Appendix 1) Summary of progress since the initial malERA initiative and remaining gaps

This table of the original malERA research and development agenda provides a convenient framework for assessing the current overall status, achievements, progress, and gaps in developing diagnostic tools for malaria elimination. Each of the topics in the original summary are reviewed below, including comments submitted by members of the malERA Refresh Panel.

| **Research area** | | | **Accomplishments since the initial malERA process** | **Refs** | **Remaining gaps** |
| --- | --- | --- | --- | --- | --- |
| Overarching questions |  | What proportion of effort should be directed to screening and surveillance versus early case detection at various time points in elimination? | The relative priorities have not been addressed, and most likely not realistically addressable or relevant, as surveillance should be considered as an independent need in its own right. |  |  |
|  |  | Do we need microscopy for elimination, or can other tests replace it? | WHO guidelines published in 2015 recommend the use of RDTs (rapid diagnostic tests) and microscopy in all transmission settings. Literature over the past recent years suggests that microscopy as currently practiced will not provide the sensitivity required to make active case detection useful nor surveillance accurate enough to monitor progress towards elimination. Other tests (starting with rapid diagnostic tests (RDTs)) can replace microscopy in many settings. | *Bousema, 2014, Escalante, 2015, Golassa, 2015, Tadesse, 2015,*  *World Health Organisation, 2015,* | How easy it will be to replace microscopy with a non-microscopic method in places with strong tradition of using microscopy remains an open question. |
| Programmatic issues |  | Further data on thresholds of (i) parasite density likely to cause symptoms in low-transmission settings with variable or waning immunity, and (ii) transmission potential of cases with parasitemia below the threshold of microscopy and RDTs | There has been significant amount of new data published on parasite density as it pertains to symptomatic vs asymptomatic and transmission, and transmission potential below the threshold of microscopy and RDTs. | *Bousema, 2014, Escalante, 2015, Golassa, 2015, Lin Ouedraogo, 2015, Tadesse, 2015.* | The analytical component that has been missing is correlating the parasitemia thresholds to antigen concentration, which is the essential metric required to drive the development of more sensitive RDTs with current antigens. There are currently ongoing studies to address this knowledge gap. |
|  |  | Diagnostic tests for non-malarial febrile illness in malaria-endemic and malaria-elimination settings | This is only relevant in the context of sustaining malaria diagnosis in malaria elimination settings. Increasing data are published on non-malarial febrile etiology. | *Mayxay, 2013; Mueller, 2014.* | There are little data published on diagnostic approaches to inform management of non-malarial febrile etiology. |
|  |  | Distribution of severe G6PD variants | Progress here includes WHO policy statements on low-dose primaquine, greater recognition that G6PD deficiency is a barrier to increasing adoption of a P. *vivax* radical cure with high dose primaquine, and the availability of a new G6PD RDT (AccessBio Carestart G6PD test). There has been a surge in number of G6PD surveys either in their own right or in combination with evaluation of the new G6PD test. | *Monteiro, 2014.* | A question arises on whether, for programmatic purposes, we need to know the distribution of severe G6PD variants (to inform malaria elimination) or just know whether it is present in a given population at risk? |
| Technical issues: case-management tools | High priority. Stable tests for case management in low-training, low technology settings with sensitivity sufficient for community level case management, including: | Greater consistency of antigen-detecting RDTs in P. *falciparum* detection, particularly in the case of non-persistent antigens | Not sufficient progress. |  |  |
|  |  | More sensitive and stable tests (antigen-detecting RDTs) to detect non-P. *falciparum* parasites | Much remains to be done to improve field tests for P. *vivax*. |  |  |
|  |  | Clarification of the programmatic/implementation requirements for antigen-detecting RDTs that will ensure good impact in the field | This is still work in progress; efforts such as the PMI funded Malaria Care are seeking to address this. | *http://malariacare.org* |  |
|  |  | Standardized low-cost positive controls for antigen detecting RDTs suitable for field use | Positive control methods for RDTs are under development (FIND and partners). PCWs can be used to test and compare RDTs; across suppliers. The PCWs have better stability as they are distributed in dry form. | *Lon, 2005* |  |
|  |  | RDTs suitable for field use | Efforts to develop more robust and sensitive RDTs are underway. Currently the IDT project works on improved detection of HRP2. Other Pf antigens should be considered to cover areas of hrp2-gene deletion See under “surveillance tools” below.  Newly deployed RDT readers were deployed in 2012, the Fio-net and the Holomic Rapid Diagnostic Reader in 2013. Both are characterized by improved cloud access and analytical software. |  |  |
|  |  | Sustainable tools for quality control of RDTs at a country level | While most RDT blood tests are easy to use for personnel with minimal training, demonstrated shelf-life and reliability vary in WHO-sponsored product tests. Following the most recent of these (round 5, 2013) two products were no longer recommended. Interchangeability of RDTs would be helped by a harmonized set of labeling, packaging and instructions. The Roll Back Malaria Partnership Harmonization Group has recently made recommendations in these areas but their implementation is uncertain. | *World Health Organisation, 2013* |  |
|  |  | Further investigation of non-blood sampling to determine the potential for detecting recoverable antigen in these samples. | There continues to sporadic evaluations of the use of saliva and urine, for both PCR and antigen detection tests for malaria markers with commercial kits available for both: urine and PCR (Norgen Bioteck Corp) and urine and a dipstick (Fyodor Biotechnologies Corp). A recent study identified volatile (breath) markers in Plasmodium *falciparum*-infected volunteers. Importantly these individuals carry low parasite densities, and the diagnostic procedure, if validated, is non-invasive. Another noninvasive approach is followed by Fyodor Biotechnologies, in late clinical stages for a urine-based dipstick which is based on pan-Plasmodium HRP2- detection. | *Berna, 2015*  Oguonu, 2014 |  |
|  |  | More consistent, reliable staining methods for microscopy | Overall, progress in this area has been limited. Leishman staining was recently evaluated as superior to Giemsa for both thick and thin smear-based Plasmodium detection, and paper microfluidic cartridge for the automated staining with acridine orange prior to microscopy was presented, as well as the use of 'scratched’ slides for thick films. | Sathpathi, 2014, Horning, 2014, Norgan, 2013 | Microscopy would be improved and standardized if simple methods for smear preparation were also developed (not only staining). |
|  |  | G6PD deficiency mapping and identification (if 8-aminoquinolones are to be used) | See text above and also drugs appendix. Given recent WHO recommendations relative to the requirement for a G6PD field deployable test this is lower priority. |  |  |
|  | Medium priority | Multiplexing: Other diseases, markers of severity | Overall there has been little progress, and this area is now of less relevance to the malaria elimination agenda. A number of tests have been described in this area, including an integrated nucleic-acid-based lab-on-chip for rapid identification and simultaneous differentiation of tropical pathogens including Pf/Pv, Dengue and Chikungunya viruses. In addition PCR-based approaches were described for the simultaneous detection of multiple Plasmodium species and/or stages, or drug-resistant parasites. | *Tan, 2014,*  *Fuehrer, 2012, Kuamsab, 2012,*  *Barnadas, 2011* | It remains to be seen if these tests can be deployed on larger scale and be suitable for field use. |
|  |  | Field G6PD detection (may be more important if tafenoquine approved), or raised priorities for P. *vivax* relapse prevention | Recent WHO recommendations and the P. *vivax* technical brief in which P. *vivax* relapse prevention has been prioritized has increased the priority of this research agenda item. There has been significant progress (also see drugs appendix):  AccessBio has made commercially available two point-of-care tests for G6PD deficiency:  The CareStart™ G6PD RDT, a qualitative test based on the lateral flow platform. There is now growing data regarding the performance of this test under controlled conditions. More data is required in operational conditions.  PATH and GlaxoSmithKline (GSK) are collaborating to accelerate the development of tests for G6PD deficiency. These tests will help support implementation of treatment using primaquine and also tafenoquine, an investigational 8-aminoquinoline-based drug that is currently under development. | *Bennett, 2013, Marcsisin, 2014, Vuong, 2015a,*  *Vuong, 2015b* | A possible with primaquine (possibly tafenoquine as well) is the recent finding that carriers of a relatively common genetic variant of P450 essentially gain no benefit from primaquine. |
|  |  | Tools to standardize and improve microscopy interpretation | In the context of elimination, microscopy could be seen as an interesting surveillance tool at reference laboratories. Using standard slide preparation and high throughput screening equipment could improve the limit of detection. This would not be limited to malaria.  Recent research exploits the availability of smartphones and light emitting diodes (LEDs) (recently reviewed in), including: the ‘foldscope’ project for ultra-low-cost microscopes; the improvement of CellScope an add-on lens that can be mounted on smartphones; Lifelens and LUCAS are other examples of apps and smartphone-compatible lenses to detect malaria. Progress is also being made towards improved algorithms such as the Coulter® 3-D VCS (Volume, Conductivity and Scatter) Technology, however these solutions require dedicated and costly hardware. | *Chu, 2015,*  *Cybulski, 2014,*  *Breslauer, 2009,*  *Phillips, 2015* | Microscopy is reliable when performed by expert personnel, but requires equipment and sample preparation. There are continued efforts to develop tools that will improve microscopy interpretation, but to date these fail to meet realistic complexity and price gaps whilst maintaining the performance criteria. |
|  | Low priority | Hypnozoite detection (becomes a high priority if feasibility can be demonstrated through further research on hypnozoite biology, identifying good biomarkers) | This is an extremely challenging topic; some experts suggest that relapse may arise both from hypnozoites and quiescent merozoites; either way, their numbers will be small and they are metabolically inert, making detection difficult. We could find no progress in this area over the past few years. | *Markus, 2012* |  |
| Technical issues: surveillance tools | High priority | Field-applicable tools for detection of low-density parasitemia in a high-throughput manner, suitable for surveys and active detection of parasite carriage in time to allow management of positive cases | WHO guidelines call for more sensitive diagnostic assays to be used in low-transmission areas. Various types of screen-and-treat programmes are being conducted in low-transmission areas to identify and eliminate local foci of transmission as. However, there is now abundant evidence that, even in low-transmission areas, a high proportion of malaria infections are not detected by microscopy or rapid diagnostic tests (RDTs), reducing the efficacy of screen-and-treat programmes. However, the extent to which more sensitive assays contribute to eradication can be debated.  The PATH DIAMETER (Diagnostics for Malaria Elimination towards Eradication) project has been working to build consensus on the technical specifications for elimination diagnostics and has published a target product profile for an infection detection lateral flow immunoassay (LFIA), aimed at delivering a highly sensitive assay for HRP2 ELISA by 2017.  In 2012, the LAMP (loop mediated isothermal amplification) Malaria Diagnostic Kit (Eiken Chemical LTD, FIND) was launched which does not require a thermocycler. This has been field-tested in a number of research studies but, as far as we are aware, not yet deployed in any routine malaria elimination programme.  The potential for point-of-care pathogen genotyping has now been demonstrated by the wide roll-out of the tuberculosis Xpert® system. PCR-based assays aimed at optimizing treatment for malaria might await a better understanding of the genetic basis of Plasmodium resistance to justify the costs and efforts.  PCR-based systems provide highly sensitive assays, The challenges are portability and suitability for field use. Approaches to make PCR assays simpler and mobile are being explored. Such assays are likely to be more expensive than microscopy or a conventional RDT and whether they are only needed for a relatively short period in the elimination effort has not yet been explored.  Serology has been tested extensively during the past few years to measure transmission intensity in different epidemiological situations and to measure the impact of control measures. The specificity of this approach has been advanced by selection of novel antigens, rather than the conventional MSP119 and AMA1 antigens used previously, and this is an approach that needs to be explored further. | *Imwong, 2015,*  *Singh, 2013,*  *Cook, 2015,*  *Dinzouna-Boutamba, 2014,*  *Yongkiettrakul, 2014, Helb, 2015,*  *Tietje, 2014* |  |
|  |  | Tools for minimally invasive, very rapid detection of low density parasite infections suitable for screening of migrants/travelers | The Infection Detection Test (IDT) initiative is seeking to develop more sensitive RDTs for low-density parasite infection detection, primarily for active case detection but could apply to this agenda item too.  Another highlight for this type of diagnostic test, under development, is the Rapid Assessment Malaria portable hemozoin detection system, which is undergoing field trials. Work has stopped on the Dark Field Cross Polarization (DFxP) hemozoin detection platform following the observation that ring-stage parasites do not contain hemozoin.  The magneto-optical Technology (MOT); it is one of the few non-invasive assays under development. The other non-invasive assay, still in lab stage, involves a microfluidic device for a label-free and non-invasive cell-counting assay through electric impedance sensing.  Progress has also been made in spectroscopic approaches, including Spectraphone and SpectraWave and SpectraNet. Both are being developed as highly portable solutions. Finally, flow cytometry was recently assessed under field conditions. | *Delahunt, 2014,*  *Mens, 2010; Orban, 2014,*  *Du, 2013,*  *E. Lukianova-Hleb, 2015,*  *E. Y. Lukianova-Hleb, 2014a,*  *E. Y. Lukianova-Hleb, 2014b,*  *Woodrow, 2015* |  |
|  | Innovation with potential for major operational impact | Noninvasive, low-density parasite detection | See above section on field-applicable tools for detection of low-density parasitemia, |  |  |
| Low-hanging fruit with immediate application for elimination |  | High-throughput field molecular detection, capable of use at district level or below |  |  |  |
|  |  | Positive control methods for RDTs | See the positive control wells described above |  | Positive controls should be added to any new diagnostics (RDT or molecular methods). More than a research and development priority, this should be a requirement to manufacturers through the WHO Prequalification department. |

**References**

Barnadas, C., Kent, D.*, et al.* (2011). *A new high-throughput method for simultaneous detection of drug resistance associated mutations in Plasmodium vivax dhfr, dhps and mdr1 genes*. *Malar J, 10*, 282.

Bennett, J. W., Pybus, B. S.*, et al.* (2013). *Primaquine failure and cytochrome P-450 2D6 in Plasmodium vivax malaria*. *N Engl J Med, 369*(14), 1381-1382.

Berna, A. Z., McCarthy, J. S.*, et al.* (2015). *Biomarkers of infection with Plasmodium falciparum detected in human breath*. *J Infect Dis*.

Bousema, T., Okell, L., Felger, I., & Drakeley, C. (2014). *Asymptomatic malaria infections: detectability, transmissibility and public health relevance*. *Nat Rev Microbiol, 12*(12), 833-840.

Breslauer, D. N., Maamari, R. N.*, et al.* (2009). *Mobile phone based clinical microscopy for global health applications*. *PLoS One, 4*(7), e6320.

Chu, K., Smith, Z. J., & Wachsmann-Hogiu, S. (2015). *Development of inexpensive blood imaging systems: where are we now?* *Expert Rev Med Devices*, 1-15.

Cook, J., Aydin-Schmidt, B.*, et al.* (2015). *Loop-mediated isothermal amplification (LAMP) for point-of-care detection of asymptomatic low-density malaria parasite carriers in Zanzibar*. *Malar J, 14*, 43.

Cybulski, J. S., Clements, J., & Prakash, M. (2014). *Foldscope: origami-based paper microscope*. *PLoS One, 9*(6), e98781.

Delahunt, C., Horning, M. P.*, et al.* (2014). *Limitations of haemozoin-based diagnosis of Plasmodium falciparum using dark-field microscopy*. *Malar J, 13*, 147.

Dinzouna-Boutamba, S. D., Yang, H. W.*, et al.* (2014). *The development of loop-mediated isothermal amplification targeting alpha-tubulin DNA for the rapid detection of Plasmodium vivax*. *Malar J, 13*, 248.

Du, E., Ha, S.*, et al.* (2013). *Electric impedance microflow cytometry for characterization of cell disease states*. *Lab Chip, 13*(19), 3903-3909.

Escalante, A. A., Ferreira, M. U.*, et al.* (2015). *Malaria Molecular Epidemiology: Lessons from the International Centers of Excellence for Malaria Research Network*. *Am J Trop Med Hyg, 93*(3 Suppl), 79-86.

Fuehrer, H. P., Stadler, M. T.*, et al.* (2012). *Two techniques for simultaneous identification of Plasmodium ovale curtisi and Plasmodium ovale wallikeri by use of the small-subunit rRNA gene*. *J Clin Microbiol, 50*(12), 4100-4102.

Golassa, L., Baliraine, F. N.*, et al.* (2015). *Microscopic and molecular evidence of the presence of asymptomatic Plasmodium falciparum and Plasmodium vivax infections in an area with low, seasonal and unstable malaria transmission in Ethiopia*. *BMC Infect Dis, 15*(1), 310.

Helb, D. A., Tetteh, K. K.*, et al.* (2015). *Novel serologic biomarkers provide accurate estimates of recent Plasmodium falciparum exposure for individuals and communities*. *Proc Natl Acad Sci U S A, 112*(32), E4438-4447.

Hopkins, H., Gonzalez, I. J.*, et al.* (2013). *Highly sensitive detection of malaria parasitemia in a malaria-endemic setting: performance of a new loop-mediated isothermal amplification kit in a remote clinic in Uganda*. *J Infect Dis, 208*(4), 645-652.

Horning, M. P., Delahunt, C. B.*, et al.* (2014). *A paper microfluidic cartridge for automated staining of malaria parasites with an optically transparent microscopy window*. *Lab Chip, 14*(12), 2040-2046.

Imwong, M., Hanchana, S.*, et al.* (2014). *High-throughput ultrasensitive molecular techniques for quantifying low-density malaria parasitemias*. *J Clin Microbiol, 52*(9), 3303-3309.

Imwong, M., Nguyen, T. N.*, et al.* (2015). *The epidemiology of subclinical malaria infections in South-East Asia: findings from cross-sectional surveys in Thailand-Myanmar border areas, Cambodia, and Vietnam*. *Malar J, 14*(1), 381.

Kuamsab, N., Putaporntip, C., Pattanawong, U., & Jongwutiwes, S. (2012). *Simultaneous detection of Plasmodium vivax and Plasmodium falciparum gametocytes in clinical isolates by multiplex-nested RT-PCR*. *Malar J, 11*, 190.

Lin Ouedraogo, A., Goncalves, B. P.*, et al.* (2015). *Dynamics of the Human Infectious Reservoir for Malaria Determined by Mosquito Feeding Assays and Ultrasensitive Malaria Diagnosis in Burkina Faso*. *J Infect Dis*.

Lindblade, K. A., Steinhardt, L.*, et al.* (2013). *The silent threat: asymptomatic parasitemia and malaria transmission*. *Expert Rev Anti Infect Ther, 11*(6), 623-639.

Lon, C. T., Alcantara, S.*, et al.* (2005). *Positive control wells: a potential answer to remote-area quality assurance of malaria rapid diagnostic tests*. *Trans R Soc Trop Med Hyg, 99*(7), 493-498.

Lucchi, N. W., Demas, A.*, et al.* (2010). *Real-time fluorescence loop mediated isothermal amplification for the diagnosis of malaria*. *PLoS One, 5*(10), e13733.

Lukianova-Hleb, E., Bezek, S.*, et al.* (2015). *Transdermal Diagnosis of Malaria Using Vapor Nanobubbles*. *Emerg Infect Dis, 21*(7), 1122-1127.

Lukianova-Hleb, E. Y., Campbell, K. M.*, et al.* (2014a). *Hemozoin-generated vapor nanobubbles for transdermal reagent- and needle-free detection of malaria*. *Proc Natl Acad Sci U S A, 111*(3), 900-905.

Lukianova-Hleb, E. Y., & Lapotko, D. O. (2014b). *Malaria theranostics using hemozoin-generated vapor nanobubbles*. *Theranostics, 4*(7), 761-769.

Marcsisin, S. R., Sousa, J. C.*, et al.* (2014). *Tafenoquine and NPC-1161B require CYP 2D metabolism for anti-malarial activity: implications for the 8-aminoquinoline class of anti-malarial compounds*. *Malar J, 13*, 2.

Markus, M. B. (2012). *Dormancy in mammalian malaria*. *Trends Parasitol, 28*(2), 39-45.

Mayxay, M., Castonguay-Vanier, J.*, et al.* (2013). *Causes of non-malarial fever in Laos: a prospective study*. *Lancet Glob Health, 1*(1), e46-54.

Mens, P. F., Matelon, R. J.*, et al.* (2010). *Laboratory evaluation on the sensitivity and specificity of a novel and rapid detection method for malaria diagnosis based on magneto-optical technology (MOT)*. *Malar J, 9*, 207.

Monteiro, W. M., Val, F. F.*, et al.* (2014). *G6PD deficiency in Latin America: systematic review on prevalence and variants*. *Mem Inst Oswaldo Cruz, 109*(5), 553-568.

Mueller, T. C., Siv, S.*, et al.* (2014). *Acute undifferentiated febrile illness in rural Cambodia: a 3-year prospective observational study*. *PLoS One, 9*(4), e95868.

Mukanga, D., Tiono, A. B.*, et al.* (2012). *Integrated community case management of fever in children under five using rapid diagnostic tests and respiratory rate counting: a multi-country cluster randomized trial*. *Am J Trop Med Hyg, 87*(5 Suppl), 21-29.

Norgan, A. P., Arguello, H. E.*, et al.* (2013). *A method for reducing the sloughing of thick blood films for malaria diagnosis*. *Malar J, 12*, 231.

Odaga, J., Sinclair, D.*, et al.* (2014). *Rapid diagnostic tests versus clinical diagnosis for managing people with fever in malaria endemic settings*. *Cochrane Database Syst Rev, 4*, CD008998.

Oguonu, T., Shu, E.*, et al.* (2014). *The performance evaluation of a urine malaria test (UMT) kit for the diagnosis of malaria in individuals with fever in south-east Nigeria: cross-sectional analytical study*. *Malar J, 13*, 403.

Orban, A., Butykai, A.*, et al.* (2014). *Evaluation of a novel magneto-optical method for the detection of malaria parasites*. *PLoS One, 9*(5), e96981.

Phillips, Z. F., D'Ambrosio, M. V.*, et al.* (2015). *Multi-Contrast Imaging and Digital Refocusing on a Mobile Microscope with a Domed LED Array*. *PLoS One, 10*(5), e0124938.

Sathpathi, S., Mohanty, A. K.*, et al.* (2014). *Comparing Leishman and Giemsa staining for the assessment of peripheral blood smear preparations in a malaria-endemic region in India*. *Malar J, 13*, 512.

Seidenberg, P. D., Hamer, D. H.*, et al.* (2012). *Impact of integrated community case management on health-seeking behavior in rural Zambia*. *Am J Trop Med Hyg, 87*(5 Suppl), 105-110.

Singh, R., Savargaonkar, D., Bhatt, R., & Valecha, N. (2013). *Rapid detection of Plasmodium vivax in saliva and blood using loop mediated isothermal amplification (LAMP) assay*. *J Infect, 67*(3), 245-247.

Tadesse, F. G., Pett, H.*, et al.* (2015). *Submicroscopic carriage of Plasmodium falciparum and Plasmodium vivax in a low endemic area in Ethiopia where no parasitaemia was detected by microscopy or rapid diagnostic test*. *Malar J, 14*, 303.

Tamiru, A., Boulanger, L.*, et al.* (2015). *Field assessment of dried Plasmodium falciparum samples for malaria rapid diagnostic test quality control and proficiency testing in Ethiopia*. *Malar J, 14*, 11.

Tan, J. J., Capozzoli, M.*, et al.* (2014). *An integrated lab-on-chip for rapid identification and simultaneous differentiation of tropical pathogens*. *PLoS Negl Trop Dis, 8*(7), e3043.

Tanner, M., Greenwood, B.*, et al.* (2015). *Malaria eradication and elimination: views on how to translate a vision into reality*. *BMC Med, 13*(1), 167.

Taylor, S. M., Juliano, J. J.*, et al.* (2010). *High-throughput pooling and real-time PCR-based strategy for malaria detection*. *J Clin Microbiol, 48*(2), 512-519.

Thiam, S., Thior, M.*, et al.* (2011). *Major reduction in anti-malarial drug consumption in Senegal after nation-wide introduction of malaria rapid diagnostic tests*. *PLoS One, 6*(4), e18419.

Tietje, K., Hawkins, K.*, et al.* (2014). *The essential role of infection-detection technologies for malaria elimination and eradication*. *Trends Parasitol, 30*(5), 259-266.

UNITAID. (February 2015). *Malaria Diagnostics Landscape Update*.

Vuong, C., Xie, L. H.*, et al.* (2015a). *Differential CYP 2D Metabolism Alters Tafenoquine Pharmacokinetics*. *Antimicrob Agents Chemother*.

Vuong, C., Xie, L. H.*, et al.* (2015b). *Differential cytochrome P450 2D metabolism alters tafenoquine pharmacokinetics*. *Antimicrob Agents Chemother, 59*(7), 3864-3869.

Woodrow, C. J., Wangsing, C.*, et al.* (2015). *A comparison between flow cytometry, microscopy and lactate dehydrogenase ELISA for Plasmodium falciparum drug-susceptibility testing under field conditions*. *J Clin Microbiol*.

World Health Organisation. (2013). *Malaria Rapid Diagnostic Test Performance*. *Results of WHO product testing of malaria RDTs: Round 5 (2013)*.

World Health Organisation. (2015). *Guidelines for the Treatment of Malaria, third edition*. [*http://www.who.int/malaria/publications/atoz/9789241549127/en/*](http://www.who.int/malaria/publications/atoz/9789241549127/en/).

Yongkiettrakul, S., Jaroenram, W.*, et al.* (2014). *Application of loop-mediated isothermal amplification assay combined with lateral flow dipstick for detection of Plasmodium falciparum and Plasmodium vivax*. *Parasitol Int, 63*(6), 777-784.

Yukich, J. O., Bennett, A.*, et al.* (2012). *Reductions in artemisinin-based combination therapy consumption after the nationwide scale up of routine malaria rapid diagnostic testing in Zambia*. *Am J Trop Med Hyg, 87*(3), 437-446.

Zimmerman, P. A., & Howes, R. E. (2015). *Malaria diagnosis for malaria elimination*. *Curr Opin Infect Dis*.

Zurovac, D., Githinji, S.*, et al.* (2014). *Major improvements in the quality of malaria case-management under the "test and treat" policy in Kenya*. *PLoS One, 9*(3), e92782.
