# Supplementary material for: malERA: An updated research agenda for diagnostics, drugs, vaccines, and vector control in malaria elimination and eradication
Source: PLoS Med. 2017 Nov 30;14(11):e1002455. doi: 10.1371/journal.pmed.1002455 (PMC5708606; doi:10.1371/journal.pmed.1002455)
Supplement: S2 Text — (DOCX) [file pmed.1002455.s005.docx]

**Drugs**

Appendix 2) Summary of progress since the initial malERA initiative and remaining gaps

This table of the original malERA research and development agenda provides a convenient framework for assessing the current overall status, achievements, progress, and gaps in developing drugs as tools for elimination. Each of the three topics in the original summary are reviewed below, including comments submitted by members of the malERA Refresh tools for elimination Panel.

| **Research area** | | | **Accomplishments since the initial malERA process** | **Refs** | **Remaining gaps** |
| --- | --- | --- | --- | --- | --- |
| Knowledge gaps and research priorities for optimizing current drugs |  | Pharmacology studies to optimize dosing regimens of 8-aminoquinolines for gametocytocidal and anti-relapse efficacy and safety | Single encounter drugs (SERCaP) under development.  Blood-stage challenge model for PK/PD work has been used to determine effects on transmission.  Anti-relapse effects can be studied in validated phase II studies or in soldier models.  There is a molecular marker correlating with decrease in parasite reduction rates clinically.  A quantitative G6PD diagnostic assay is under development.  Primaquine combinations with new ACTs has been studied.  Four new compounds have entered phase II trials against blood stages of malaria, and some of these have activities against liver stages.  Clinical development of tafenoquine | *Bennett, 2013; Hanboonkunupakarn, 2014; Jittamala, 2015; Marcsisin, 2014; Pukrittayakamee, 2014. Llanos-Cuentas A, Lacerda MV, Rueangweerayut R, Krudsood S, Gupta SK, Kochar SK, Arthur P, Chuenchom N, Möhrle JJ, Duparc S, Ugwuegbulam C, Kleim JP, Carter N, Green JA, Kellam L.*  *Lelievre, 2012; Lucantoni, 2013; McCarthy, QIMR*  *Lancet. 2014 phase II data on tafenoquine* | PK of primaquine in combination with artemether-lumefantrine (that may inhibit primaquine metabolism).  Impact of CYP2D6 on exposures and relevance for gametocytocidal activity.  No new NCEs with activity against the relapses of P. vivax.  Maintaining P vivax hypnozoites in human cells in culture needs reinforcing.  Current primate models are not robust enough for drug screening. |
|  |  | Rapid and robust point-of-care glucose-6-phosphate dehydrogenase (G6PD) test to improve safety of 8-aminoquinoline use | Two point-of-care tests for G6PD deficiency commercially available. | *Roca-Feltrer, 2014. Adu-Gyasi, 2015; Kim, 2011. Eziefula, 2014b* | Understanding the G6PDd genotypes (more than 440 G6PD gene variations have been identified) phenotypes and varying severity of enzyme activity deficiencies G6PDd. |
|  |  | Tests that can detect resistance to artemisinins and ACT partner drugs  (See also malERA Refresh paper ‘Insecticide and drug resistance’.) | In the Greater Mekong Subregion slow rates of P. *falciparum* parasite clearance following treatment with artesunate are now known to be associated with mutations in the PF3D7 Kelch propeller domain.  Piperaquine resistance is associated with Pfmdr1 copy number variation.  Molecular markers for resistance to ACT partner drugs amodiaquine and mefloquine are well understood. | *F. Huang, 2015, Tun, 2015, Mok, 2014, Straimer, 2014, Ariey, 2014; Ashley, 2014.* | Understanding how quickly the Kelch13 mutations and piperaquine resistance will move to Africa, and the impact of this on malaria in hyperendemic regions.  Diagnostic tools to detect resistant parasites which can be used in the field to guide optimal treatment.  Further testing of triple therapy combinations. |
|  |  | Determine gametocytocidal and anti-relapse activity of current drugs and those in the pipeline | Great progress in terms of assays, drugs and human challenge studies for transmission-blocking activity.  A blood stage challenge model for PK/PD work is established.  There are better models for P vivax, e.g. humanized mouse.  Higher through-put assays for gametocytes have been developed  Assays to measure transmission blocking activity have been developed.  Human challenge studies: Solved for drugs against blood stage parasites and pre-erythrocytic activity. | *McCarthy, QIMR; Bolscher, 2015; M. Delves, 2012; Ruecker, 2014; Stone, 2014; Baragaña, 2015; McNamara, 2013; Phillips, 2015; Marquart, 2015a, 2015b.* | Human challenge studies to assess transmission-blocking effects.  Human challenge studies to assess anti-hypnozoite activity.  Human challenge studies to assess anti-relapse.  Relationship between in vitro assays on transmission blocking potential and transmission reduction in vivo  Relevance of differential effect of drugs on male/female gametocytes and transmission in natural conditions  Assays to measure transmission blocking activity clinically. |
| Knowledge gaps and research priorities for developing new drugs for malaria eradication | Desired products | Drugs that prevent transmission by killing or preventing development of gametocytes, or blocking sporozoite development in the mosquito | Standard Membrane Feeding Assay to test the ability of all development candidates are completed.  Approved drugs of this kind are low dose primaquine for its anti-gametocyte activity, or ivermectin for its effect as an endectocide.  Optimal ivermectin doses are being investigated.  Other drugs are progressing in the pipeline, with some in phase II (KAE609, phase I, SJ733 or in preclinical development, DDD107498).  There has been progress in the development of new phenotypic screens involving gametocytes  Positive results of transmission-blocking properties are seen with new antimalarial molecules targeting PfATP4, such as KAE609 and SJ733, and with those targeting PfEF2 such as DDD498. | *Dantzler, 2015; Douglas, 2013; Duffy, 2013; Wu, 2015, Lelievre, 2012, Lucantoni, 2013, Graves 2015* | The effect of low dose primaquine in addition to the treatment regimen for clinical P. *falciparum* malaria on transmission at the community level and needs to be evaluated  With the availability of new gametocyte assays a number of studies have tested whether existing antimalarials target gametocytes, and the  In P. vivax infections, new drugs which prevent relapses are needed.  The relation between plasma exposure and transmission blockade is not well modelled at this stage. |
|  |  | Drugs that cure liver stages of vivax (and ovale) malaria | (See in this table ‘Assays to measure activity against liver stages’) |  | Need drugs with no hemolytic risks to prevent relapse from P. vivax or P ovale. |
|  |  | Ideally, drugs that can be administered in a single encounter at infrequent intervals, and that result in radical cure of all parasite stages | There has been significant progress in this area. There is a stronger pipeline of new compounds, with several having reached phase II over the last five years. | TNC Wells 2015 | Further identification of new chemical entities is needed to maintain the pipeline sufficiently. |
|  |  | Sustained or pulsed release formulations |  |  | Injectable formulations of medicines which could be used to provide long term protection are needed.  Need new regimens of drugs that could be given as a single injection in a low volume.  More potent drugs are needed |
|  |  | Exceptionally safe schizonticidal drugs for curing asymptomatic falciparum infection |  |  | There is little understanding of the appropriateness of current therapies (ACTs) for treating asymptomatic infections, and the risk:benefit for the individual needs to be determined clinically.  There is no accepted definition of what proportion of the asymptomatic carrier population would need to be effectively treated to have a significant effect on transmission  New chemical entities are needed for use in combination therapy of uncomplicated asymptomatic malaria |
|  | Fundamental research questions aimed towards developing desired drugs | Fundamental studies of liver and sexual stage biology (in both host and mosquito) | (See malERA Refresh paper ‘Basic science and enabling technologies’.) |  |  |
|  |  | Mechanisms of resistance and pharmacological strategies to deter resistance  (See also ‘Tests that can detect resistance ‘ in this Table above and malERA Refresh paper ‘Basic science and enabling technologies’.) | Significant progress has been made in understanding the molecular basis of drug resistance.  A wide variety of different resistance mechanisms have been shown, some of which have linked directly back to molecular targets.  There are several molecules against which resistance cannot be raised in culture using parasite levels of in excess of 109.  Clinical work in the Greater Mekong Subregion shows that the parasite may not be able to sustain resistance against two 4-aminoquinolines simultaneously, | *E Winzeler et al., unpublished. TRAC2 study 2016/7.* | Further testing of combinations (4-aminoquinolines) is needed. |
|  |  | In vitro culture of P. vivax to understand parasite biology  (See also malERA Refresh paper ‘Basic science and enabling technologies’.) | An improved cell culture system for P.*vivax* has been reported.  Recent work using spatial restriction of primary human hepatocytes, or use of a human hepatocyte line HC04, can be used to demonstrate infection with primary P. *vivax* sporozoites, and it appears that these infections can result in dormant forms. | *Roobsoong, 2015, personal communications Dennis Kyle, Sangheta Bhatia and Jetsumon Sattabongkot Prachumsri* | Cellular assays of P. *vivax* dormant stages in human hepatocytes are needed to allow drugs to be screened.  P.*vivax* activity assays need to be standardized between research groups |
|  | Tools and capacities | Increased capacity for clinical pharmacology research including pharmacokinetics/pharmacodynamics studies in populations targeted for malaria elimination |  |  |  |
|  |  | Increased capacity for human challenge studies for early go/no go decisions on drug candidates | Growing capacity and utilization of the controlled human malaria infection (CHMI) model; new antimalarial drug candidates are now almost systematically tested in Plasmodium falciparum -infected volunteers in order to acquire precise PK/PD parameters in immuno-naïve individuals. | *Engwerda, 2012; Marquart, 2015a; McCarthy, 2013; McCarthy, 2011; McCarthy, 2014a; McCarthy, 2014b; Stanisic, 2015* |  |
|  |  | Assays to measure transmission-blocking activity |  |  |  |
|  |  | Assays to measure activity against liver stages | Progress in the development of cell biology models for liver stage infection. Assay systems include spatially restricted human primary hepatocytes and specifically selected HC04 cell line. |  | The assays are limited by a simple supply of P vivax sporozoites |
|  |  | In vitro culture of P. vivax and other non-falciparum species for drug screening | All new medicines are routinely screened against primary clinical isolates from other non-falciparum strains  No progress on a screening assay for P.*vivax* or other non-falciparum species. | *Zeeman, 2013* |  |
|  |  | Genomic and proteomic approaches to identify transmission- blocking and liver-stage activity |  |  | There is still a need to identify circulating biomarkers for the presence of hypnozoites. |
| Knowledge gaps and research priorities for drug treatment and prevention strategies for eradication |  | Field studies to evaluate new drugs and approaches in a variety of epidemiological settings | *WARRN and MDA stuff* |  |  |
|  |  | Robust and highly sensitive malaria diagnostics for malaria infection and especially for carriage of infectious gametocytes  (See also malERA Refresh appendix ‘Diagnostics’.) | Progress has been made in gametocyte detection using Pfs25 PCR and NASBA  New qPCR targets have been described, permitting the ultra-sensitive detection of P. *falciparum* by amplification of multi-copy sub-telomeric targets.  Ultrasensitive diagnositic approaches have been field-tested including a PCR-based lab-on-a-chip platform, high-throughput, colorimetric, field applicable loop-mediated isothermal amplification (HtLAMP) assay | *Slater, 2015, in press*  *Britton, 2015*  *Hofmann, 2015*  *Lin Ouedraogo, 2015.*  *Koepfli, 2015; Mwingira, 2014; Walker, 2015; Wampfler, 2013; Taylor, 2014* | Determine how sensitive next-generation rapid diagnostic tests need to be for use in Plasmodium falciparum malaria elimination.  Sensitivity and specificity of markers for infectious gametocytes need to be improved.  Comparative sensitivity of different diagnostics |
|  |  | Measures to monitor and improve adherence and safety | Understanding of low incidence serious adverse events to current treatments has developed significantly.  Safety studies of 15’000 patients (ASAQ), and over 10’000 events (DHA-PQP, and pyronaridine artesunate). |  | Safety after repeated administration of ACTs is still to be understood |
|  |  | How must drug treatment and prevention strategies change as elimination proceeds?  (See also malERA Refresh paper ‘Combination interventions and modelling’.) | WHO recommendation on MSAT and MDA. | Okell, 2014; Slater, 2015, in press | When to switch from MDA to MSAT is unclear |
|  |  | Strategies to deter resistance  (See also malERA Refresh paper ‘Insecticide and drug resistance’.) | Progress in the testing triple combinations to overcome partner resistance (see in this table ‘Tests that can detect resistance to artemisinins and ACT partner drugs’) | TRAC2 study on WWARN website |  |
