# Supplementary material for: malERA: An updated research agenda for diagnostics, drugs, vaccines, and vector control in malaria elimination and eradication
Source: PLoS Med. 2017 Nov 30;14(11):e1002455. doi: 10.1371/journal.pmed.1002455 (PMC5708606; doi:10.1371/journal.pmed.1002455)
Supplement: S3 Text — (DOCX) [file pmed.1002455.s006.docx]

**Vaccines**

Appendix 3) Summary of progress since the initial malERA initiative and remaining gaps

In 2011 malERA proposed the concept of “Vaccines that Interrupt Malaria (Parasite) Transmission” (VIMT) that have as a primary goal interrupting parasite transmission through population-based immunization of individuals at risk for parasite infection and/or contributing to the infectious reservoir. All parts of the parasite’s lifecycle and the mosquito vector are considered potential immune targets. A single Target Product Profile (TPP) for a ‘VIMT’ was outlined with a defined indication, and, for each product characteristic, a ‘‘desired target’’ (aspirational) and a ‘‘minimally acceptable target’’ (must achieve). As part of a strategy for product and clinical development, a classification schema for vaccine program progression was proposed based on the following three stages of development: 1) preclinical feasibility studies; 2) translational projects (pre-proof-of-clinical concept); and 3) vaccine candidates (phase III through licensure).

The summary below of the 2011 research and development agenda provides a convenient framework for assessing the current overall status, achievements, progress, and gaps in developing vaccines as tools for elimination. Each of the five topics in the original summary are reviewed, including comments submitted by members of the malERA Refresh tools for elimination Panel. See the full paper for the new considerations raised by the malERA Refresh panel on tools for elimination.

| **Research area** | **Accomplishments since the initial malERA process** | **Refs** | **Remaining gaps** |
| --- | --- | --- | --- |
| Development and application of novel vaccine delivery approaches and/or adjuvants to elicit long-lasting protective efficacy that makes significant impact on malaria transmission rates under diverse epidemiological settings. | Some VMIT projects have progressed and others have been eliminated in either the preclinical or translational stages (see detailed narrative* below).  A number of new adjuvants, some of which are variants of AS01, are being evaluated for human use in preclinical feasibility studies.  Several approaches have reached high level efficacy in preventing infection in controlled human malaria infection (CHMI) models, including whole sporozoite (either radiation-attenuated (PfSPZ) or given with concurrent chemoprophylaxis) or subunit CSP (FxRTS,S) delivered with a delayed fractional third dose.  Direct venous inoculation with PfSpz has advanced to field studies; the use of PfSPZ vaccine candidate is being explored for elimination projects.  FxRTS,S is being evaluated for its role in preventing infection as a primary goal.  The IDRI portfolio of adjuvants and GSK AS series of adjuvants are being made available for studies of VIMTs.    Different platforms for preparing particles, including chemical conjugation to various carriers, are being explored, including the use of carrier proteins made available from industry partners.  Controlled human malaria infection (CHMI) models are being developed and should be quite helpful | *Seder, 2013; Gomez-Perez, 2015* | Predictive tools for the human efficacy of adjuvants are still lacking.  Late stage clinical data of efficacy in interrupting malaria parasite transmission  Feasibility of mass vaccination campaigns of whole populations with current early stage vaccine candidates  Long-lasting protective efficacy |
| Expansion of vaccine development efforts to cover Plasmodium species other than P. falciparum, especially P. vivax (including hypnozoites). | Currently only three *P. vivax* vaccines have reached clinical trials (PvDBP, PvCSP and Pvs25) and only one is likely to go forward to Phase II (see detailed narrative** below. | *Reyes-Sandoval, 2013;* | Unfortunately there has not been adequate funding to support development efforts to cover Plasmodium species other than *P. falciparum*, especially *P. vivax*. As such, progress has been slow to non-existent, particularly in the translational and vaccine candidate stages of development. |
| Understanding the dynamics between multiplication of asexual stage parasites, gametocytogenesis, and malaria transmission rates at the population level. | Various malaria models have been developed – MAP, Open Malaria, STI.  A newly developed mouse population transmission model, initially tested using an anti-malarial drug, but now being tested with vaccines/biologics, has the potential to inform the development of transmission-blocking interventions.  In addition to informing efficacy requirements as a function of transmission intensity, and thereby informing TPPs, the model may be extended to evaluate interventions targeting multiple lifecycle stages and drug/vaccine/biologic combinations to inform potential clinical studies. | *Bousema, 2011; Griffin, 2010; Blagborough, 2013.* | It remains to each field site at present to define the burden of asexual stage parasites and gametocytogenesis, and how these parameters correspond to malaria transmission rates, for their population under study.  No tools yet exist that will fully predict whether an individual in an endemic area will or will not transmit to a mosquito feeding on that individual. |
| Development of robust assays to study functional immune responses at the individual level that can predict effect on malaria transmission at the population level and allow decision making in product development. | Factors influencing transmission that are being studied include the duration of human infectiousness, frequency of sampling by mosquitoes and variation in vector competence among different mosquito populations.  Direct mosquito feeds in malaria endemic settings have been established in multiple research sites, and longitudinal studies to determine human infectiousness to mosquitoes are ongoing.  Multiple studies have been initiated comparing direct mosquito feeds to direct membrane feeds to standard membrane feeds. Initial results suggest that direct feeds may be more stringent measures of infectivity.  Work from RTS,S looking at anti-CSP this work is still ongoing and could generate useful insights.  In malaria non-immunes vaccinated with Pf SPZ vaccine (radiation-attenuated sporozoites), we have simple immunological measures (ELISA) that have a high (>90%) positive predictive value for protection. These same measures do not work well in malaria-exposed individuals; however, we have preliminary data that cell-based assays may predict protection against natural transmission in the field. This is a new area of exploration for Sanaria, but they anticipate that within 2- 3 years they will have reliable biomarkers predicting sterile immunity against infection. | *Stone, 2015; Ouédraogo, 2015; Boyle, 2015; Foquet, 2014; Behet, 2014; Bijker, 2013; Bijker, 2014; Felgner, 2013.* | Improving an understanding of which individuals transmit infections to mosquitoes has been informed by only a handful of studies that have directly determined the infectiousness of individuals in endemic populations.  More work is needed to establish the correlation between these functional assays at the individual level  The predictability of functional assays at the individual level to malaria parasite transmission at the population level remains uncertain, and as such the value of such functional assays in decision-making for product development also remains uncertain. |
| Development of tools to measure malaria transmission rates, thereby facilitating clinical development of vaccines that reduce malaria transmission. | We now have better serological tools and are making, better use of available tools e.g. gametocyte quantification, mFOI-Measuring force of infection, and transmission experiments.  A new research study has been funded to develop a transmission model based on the injection of infectious sporozoites and then to look for transmission during the subsequent period of parasitemia. This extension of the CHMI model may achieve large efficiencies in the study of transmission blocking technologies aimed not at preventing infection but at preventing transmission in those parasitemic. | *Helb, 2015; Koepfli, 2015; Lin Ouédraogo, 2015; Mueller, 2012; Tusting, 2014;Seder, 2013.* | Work is ongoing to use different measures/assays as tools to estimate transmission rates in populations however; it is not clear how these will replace traditional diagnostics in Phase 1- Phase 3 trials of transmission-blocking vaccines.  Direct skin feeds are being explored in field trials. However, these are labor and human use intensive because a large cohort of vaccine recipients has to be studied. |

**Detailed narrative and panellists’ comments:*

Pipeline overview and trends

Possibly stimulated by the success of RTS,S, the malaria vaccine pipeline includes a number of innovative candidates, although none of these have progressed beyond Phase II [Sheehy, 2013; World Health Organization, 2014]. One of the key difficulties for developing a malaria vaccine remains that, unlike viruses or bacteria, Plasmodium has plenty of genome space to vary surface antigen expression; it carries about 60 different copies of the var (Pf) or vir (Pv) family of surface genes [Guizetti, 2013; S. A. Kyes, 2007; Singh, 2014] and uses this repertoire to switch between these during a single infection to evade host immune responses [Kirkman, 2012; S. Kyes, 2007].

Part of the RTS,S success can be ascribed to its innovative adjuvant AS01, an oil-in-water emulsion that consists of MPL (mycobacterial cell wall skeleton) and QS21 (saponin derivative from the soap bark tree Quillaja saponaria; [Stoute, 1997]). A number of Phase II projects involves new experimental preparations such as QS21 (a detoxified saponin derivative; [Alving, 2012]), AS02A [Hu, 2008; Laurens, 2013; Thera, 2008], Alhydrogel® [Shimp, 2013] and GLA-SE [Coler, 2015; Patton, 2015]. However, it remains difficult to predict human efficacy (both for antigenicity and adjuvant) from animal models [Alving, 2012; Petrovsky, 2004].

The majority of experimental vaccines targets either the pre-erythrocytic stages, with CSP (circumsporozoite protein, the major surface protein of sporozoites) as antigen (like RTS,S), or the erythrocytic stages, targeting MSP1/3, (the merozoite surface protein 3), AMA-1(apical membrane antigen-1), GLURP (glutamate-rich protein) or DBP (Duffy Binding Protein; Table 6). Transmission-blocking vaccines are based on PfS25 (zygote- and ookinete-specific surface protein), Pfs230 and Pfs48/45 (gametocyte- and gamete-specific proteins) while Var2CSA-based vaccines attempt to prevent placental malaria parasite infection. With the availability of Plasmodium genomes there is now an opportunity to explore additional antigens as well [de Koning-Ward, 2015; Doolan, 2014; Duffy, 2012; Luo, 2015; Proietti, 2014].

A large number of platforms have been evaluated (often in combination). An antigen may simply consist of protein in adjuvant, sometimes expressing multiple, discontinuous antigens, and/or conjugated with a boosting (non-Plasmodium) antigen. The antigen may be expressed on virus-like particles (VLPs) generated by, e.g. AlMV (alfalfa mosaic virus), a plant virus. Antigen may also be expressed by injected adenovirus [Schuldt, 2012], e.g. the chimpanzee ChAd63 strain or human Ad26/35 serotypes, or by the Modified Vaccinia Ankara (MVA) orthopoxvirus.

Encouraging results have been obtained with whole organism-based vaccines such as PfSPZ [Seder, 2013], or attenuated P. falciparum sporozoites. This general type of vaccine successfully prevents protozoan disease in veterinary medicine [McAllister, 2014]. The strength of this approach is that a multitude of antigens is administered; moreover the elicited immune response will target the parasite infection in the bud, at low parasite density. In the veterinary setting, PfSPZ was administered 4-6 times intravenously, which also raises significant issues in terms of production, delivery and costs. Unfortunately, intramuscular administration, which is easier, appears much less effective [Gomez-Perez, 2015b].

Transmission-blocking vaccines [Kapulu, 2015; Nikolaeva, 2015] seemed to now have broken through a perceived regulatory hurdle. US FDA has opined that there is no clear legal barrier to licensing a vaccine with no direct benefits, and that the regulations do not specifically require that a biological product confer a direct clinical benefit on recipients. Acceptance of such an approach could benefit from modeling studies that demonstrate how populations in malaria-prone areas would benefit over time [Eckhoff, 2014; McCarthy, 2015; Nunes, 2013; Smith, 2012].

There has been progress in this area, with improvements in at least three vaccine technologies that have been substantively assessed in clinical testing, including field efficacy trials in Africa. All three are pre-erythrocytic stage vaccines, which would be ideal vaccines to interrupt malaria transmission (VIMTs) since they protect the recipient from clinical malaria in addition to halting transmission from that individual. The target of pre-erythrocytic stage vaccines has ranged from prevention of clinical illness to prevention of infection altogether. Until now, only the former objective could be contemplated. Now, as efficacies improve, it is possible to contemplate prevention of infection (making the vaccine a VIMT):

**A**. There has been progress in second generation RTS,S-based approaches that are showing efficacies better than 75% against CHMI, as reported in international meetings:

(1) combination with viral vectors (Hill, unpublished)

(2) delayed, fractional booster dose (Regules, unpublished).

In a review of malaria vaccines that is in press [Hoffman, 2015], co-author Johan Vekemans writes about RTS,S:

"*Efforts towards the generation of higher [RTS,S] vaccine efficacy are ongoing. Various approaches are being considered in the context of multi-institutional collaborations, including increasing immunogenicity using RTS,S/AS01-only based alternative immunization regimens [this is referring to delayed, fractional booster dose, as one possibility for alternative regimens], the use of other PfCSP-based platforms [an example would be viral vectors given in prime-boost combination], and combination of RTS,S/ AS01 with alternative antigen targets of the pre-erythrocytic [e.g., ME-TRAP from Oxford], blood or sexual stages*."

Thus second generation RTS,S-based vaccines are on their way, and promise considerably higher efficacy than the first generation vaccines. Much of this material is not published.

**B**. There is now some evidence from the field that viral vectors in heterologous prime-boost combination may have efficacy against natural transmission, although the results need to be confirmed as one published study, which showed 67% efficacy against clinical episodes in a field trial in Mali, had some unusual characteristics. Nearly all the infections occurred in the period immediately following immunization; transmission was then greatly reduced for the remainder of the short follow-up period [Ogwang, 2015]. Confirmation or lack thereof should be coming soon, from a study in Burkina Faso.

**C**. There has been progress with whole sporozoite approaches: (1) injectable radiation-attenuated sporozoites (*Pf*SPZ vaccine) have shown high level (85-100%) sterile protection against CHMI [Seder, 2013]; Seder unpublished; Epstein unpublished) including substantial protection against heterologous CHMI, and durable protection (Seder, unpublished - results to be announced at the 2015 annual meeting of ASTMH). They have also shown statistically significant sterile protection in the field, durable for 6 months (Sissoko, unpublished - results to be announced at the 2015 annual meeting of ASTMH); (2) injectable, infective sporozoites given with concurrent chemoprophylaxis (CPS) (*Pf*SPZ-CVac) have also shown high level sterile protection against CHMI durable for at least 9-10 weeks (Mordmiller, unpublished - likewise will be announced at the 2015 meeting of ASTMH), with testing of CPS in the field pending. The CPS approach has been validated in proof of concept studies using mosquito bite administration [Bijker, 2014a; Roestenberg, 2011; Schats, 2015]. In sum, efficacy figures for sterile immunity against infection (*i.e.,* meeting the criteria for VIMT), ranging from 65 to 100% against CHMI, are now being reported, in conjunction with field efficacy against infection (not clinical cases) approaching 30%. These figures were unheard of in 2011 and indicate accelerating progress. They also indicate the need for strong support of all vaccine approaches. RTS,S/AS01 has continued its important trajectory, although without second generation improvements likely has little to contribute to elimination campaigns. The phase 3 trial of RTS,S established an impact on episodes of clinical malaria in older infants and young children, but any effect on blocking infection appears to be minimal.

Clinical evaluation of several *Pf*s25-based vaccine formulations has been completed over the past few years, including an EPA conjugate and VLP fusion, both formulated with Alhydrogel. Results have generally been disappointing, suggesting new targets, or improved immunogens and/or adjuvant formulations are needed [Talaat, 2013]. The first combination SSM-TBV vaccines approach (*Pf*s25-EPA + *Pf*s230-EPA formulated on Alhydrogel) to enter clinical testing should yield results over the coming years. Targeting Pre-erythrocytic and asexual Stages:

- Regarding pre-erythrocytic vaccine approaches, significant activity has focused on improving the efficacy associated with existing vaccine candidates, notable RTS,S/AS01
- Results from a repeat of a CHMI study that was associated with high levels of protective efficacy (6/7 protected) was reported at the 2015 ASTMH meeting. The new study demonstrated that high level of efficacy from infection was associated with a delayed fractional booster dose of RTS,S.
- High levels of protective efficacy for irradiated sporozoites, delivered intravenously, have been reported in initial CHMI studies [Seder, 2013]. However, it remains to be determined whether these results can be reproduced in an endemic field setting and whether the considerable technological challenges in terms of manufacturing, formulation, and delivery, highlighted in the original malERA Vaccines manuscript, can be overcome to meet the needs of a global eradication agenda [malERA Consultative Group on Vaccines, 2011].
- Subunit vaccine strategies based primarily on induction of CD8 T cells targeting liver-stage parasites, which have been associated with low levels of protective efficacy in CHMI studies, are assessing the potential for increased breadth of immune responses via inclusion of additional antigens and testing in CHMI studies [Chuang, 2013; Ewer, 2013].
- Vaccine approaches that have failed to induce protective responses, such as CelTOS/ Alhydrogel, are being evaluated with more potent adjuvants, in this case AS01.
- Promising new asexual stage vaccine targets, such as Rh5, are advancing toward initial clinical testing, which is directly attributable to the research efforts that enhanced our understanding of the mechanisms of parasite invasion. The resultant vaccine approaches are expected to contribute primarily to prevention of disease/death, but a role in elimination cannot be ruled out.
- A large consortium based initiative, led by MVl, to assess sporozoite-stage antigens that could be targets of humeral immunity, in a systematic and consistent manner, to inform future vaccine development efforts, has been ongoing for several years.

Targeting the Sexual and Mosquito Stages: Vaccine development efforts remain focused on a relatively small number of targets (primarily *Pf*s25, *Pf*s48/45 and *Pf*s230), with only one (*Pf*s25) having advanced to clinical testing. A larger panel of target antigens on which to focus vaccine development efforts is needed.

Targeting Pre-erythrocytic and asexual Stages: Vaccine development efforts remain focused on a relatively small number of target antigens. A larger panel of target antigens on which to focus vaccine development efforts is needed. Of particular value will be antigens that can be effectively targeted with lower magnitude immune responses (humoral or cellular), toward the development of vaccines with improved durability of protection.

The concept of a vaccine directed primarily at the sexual stages of the malaria parasite is an old one and attempts at developing such a vaccine have been in progress for at least 30 years but with only very limited success. Development of such vaccines has, however, recently been given higher priority by MVI and other groups and some progress has been made but this has been slow. Attention during the past few years appears to have directed primarily at the Pfs25 P. falciparum antigen and on developing vaccine formulations, including expression in plants and algae [Jones, 2014; R. M. Jones, 2015; Jones, 2013; S. Jones, 2015; Patra, 2015], that present the an antigen in a conformation that gives high concentrations of transmission-blocking antibodies. However, progress in taking these constructs into clinical trials seems to have been slow. In summary, some progress has been made in addressing some of the research and development priorities set out in the agenda proposed five years ago by the members of Mal ERA but in some areas progress has been disappointingly slow.

In general, a large number of platforms are being experimented with (often in combination). New viral vectors (*e.g*. chimpanzee adenovirus [Dicks, 2012]) and adjuvants (*e.g.* CD74 invariant chain, [Spencer, 2014]) have been discovered. Recent experiments with expression by viral vectors in a prime boost regimen looked promising for achieving prolonged immune response [Kapulu, 2015]. Highly effective pre-erythrocytic vaccines have the potential to block transmission by reducing numbers of sexual stage parasites formed. The Sanaria group has achieved apparently sterilizing protection in a fraction of volunteers receiving intravenous immunization with cryopreserved, irradiated *P. falciparum* sporozoites [Seder, 2013], including substantial protection against heterologous CHMI, and durable protection (Seder, unpublished - results to be announced at the 2015 annual meeting of ASTMH), and is now taking that vaccine to trials in volunteers [Gomez-Perez, 2015a]. Two groups have demonstrated protection with genetically attenuated sporozoites in humanized mice [Mikolajczak, 2014; van Schaijk, 2014]. At least one of these is proceeding to clinical trial.

***Detailed narrative and panellists’ comments:*

Efforts to develop a *P. vivax* vaccine are hampered by our inability to *in-vitro* culture this species. Therefore there are only a few candidate vaccines against *P. vivax* in clinical trials [Mueller, 2015]. It is widely accepted that *P. vivax* is the most difficult to eliminate as this species leaves behind hypnozoites in hepatocytes which can trigger a relapse in the subsequent months thereby threatening malaria elimination efforts. It is evident in settings where both species exist and where effective control eliminates *P. falciparum, P. vivax* continues to persist. Vaccines against hypnozoites will get impetus when current efforts to induce sporozoite invasion of liver cell line will succeed and standard liver infection assays become available [Reyes-Sandoval, 2013]. In addition, studies focusing on major antigenic diversity of *P. falciparum* and *P. vivax* can provide important leads in selecting most common candidate antigens for vaccines that will have wider geographical acceptable efficacy [Barry, 2014; Birkett, 2013; Nunes, 2014].

The inclusion of *P. vivax* in the updated Malaria Vaccine Technology Roadmap strategic goals for malaria vaccine development efforts to prevent clinical disease and death, and transmission to accelerate eradication has been an important advancement. Previously, the Roadmap focused exclusively on the prevention of clinical disease and death in young African children caused by *P. falciparum* malaria, so this represents an important and significant shift to align global malaria vaccine development efforts with the eradication agenda. This includes understanding the dynamics between multiplication of asexual stage parasites, gametocytogenesis, and malaria transmission rates at the population level.

But overall, there has been little progress in this area. The original thinking - that general vaccine technologies successful against *P. falciparum* will likely also work against *P. vivax*, probably is largely true, with the possible exception that *P.v.* hypnozoites may be harder to eliminate than regular *P.f.* liver stages. This means that the lack of progress is not such a problem. As the vaccines against *P. falciparum* mature, the technologies can be tried for efficacy against *P. vivax* using two approaches: (1) evaluating if *Pf* vaccines cross-protect; (2) mimicking successful vaccine technologies for *Pf* but using *Pv*-derived immunogens. Particular attention has to be paid to the hypnozoite stage, which is where vaccines against *P. falciparum* might not work in translation to *P. vivax*. Transfer of technologies from *Pf* to *Pv* will be difficult because of the aforementioned difficulty in culturing the bloods stages of *Pv*. Funds should go to support the establishment of *Pv* culture capability and other tools that may facilitate the development of *Pv* vaccines. Models allowing the testing of vaccines against hypnozoites are also needed.

**Funding for *P. vivax* vaccines**

Not so much an R&D question, but a critical issue remains the chronic underfunding of *P. vivax* malaria vaccine development. The funding community has generally failed to embrace the critical need for investment in *P. vivax* in the context of malaria elimination and eradication. In a world where prevention of severe disease and death was the metric (*i.e.* pre-malERA), it was somewhat understandable that *P. vivax* took a backseat to *P. falciparum*, at least based on the disease burden data available at that time. However, in the context of elimination and eradication, where *P. vivax* is generally regarded to be the 'last parasite standing', this is no longer the case. Input from experts who have worked on other elimination/eradication efforts repeatedly (most notably polio) highlight the need to initiate work 'early' on interventions to address what are expected to be the most challenging elements; *P. vivax* elimination/eradication would fit this category.
