# Supplementary material for: malERA: An updated research agenda for diagnostics, drugs, vaccines, and vector control in malaria elimination and eradication
Source: PLoS Med. 2017 Nov 30;14(11):e1002455. doi: 10.1371/journal.pmed.1002455 (PMC5708606; doi:10.1371/journal.pmed.1002455)
Supplement: S4 Text — (DOCX) [file pmed.1002455.s007.docx]

**Vector Control**

Appendix 4) Summary of progress since the initial malERA initiative and remaining gaps

The summary below of the 2011 research and development agenda provides a convenient framework for assessing the current overall status, achievements, progress, and gaps in developing vector control tools for elimination. Each of the topics in the original summary are reviewed, including comments submitted by members of the malERA Refresh tools for elimination Panel. See the full paper for the new considerations raised by the malERA Refresh panel on tools for elimination.

| **Research area** | **Accomplishments since the initial malERA process** | **Refs** | **Remaining gaps** |
| --- | --- | --- | --- |
| Development of an analytic framework that can bring together existing and new information on all aspects of malaria and malaria transmission through a public portal designed to facilitate decision making by the malaria research, control, and tool development communities. | Publicly available information on vector control includes yearly overviews of the vector control pipeline from Innovative Vector Control Consortium (IVCC; [www.ivcc.com](http://www.ivcc.com)), useful resources from the WHO ([www.who.int/malaria/areas/vector_control/](http://www.who.int/malaria/areas/vector_control/)) and other portals such as VecNet.  There is progress in modelling of intervention strategies, population dynamics, and within-host and transmission dynamics. | *IVCC,* [*www.ivcc.com*](http://www.ivcc.com)*; WHO,* [*www.who.int/malaria/areas/vector_control/*](http://www.who.int/malaria/areas/vector_control/)*; Griffin, 2010; White, 2011; Eckhoff, 2013.* | See other papers in the malERA Refresh series. |
| An improved choice of insecticides, and formulations coupled with improved methods to reduce the risk of resistance to ensure that the availability of effective insecticides does not become the limiting factor in our ability to reduce transmission to levels where local elimination can be attempted. | The insecticide development pipeline includes nine new classes of active ingredient chemistries with modes of action different to pyrethroids.  Actellic 300CS is now being implemented in some areas.  A large number of potentially larvicidal agents, especially of biological origin are being explored.  See the paper on resistance in the malERA Refresh series. |  | See the paper on resistance in the malERA Refresh series.  There is a lack of good biological markers of resistance in mosquitoes.  There is a lack of standardized and interpretable bioassays for estimating resistance in field populations.  The relationship between the spread of insecticide-resistance and impact on malaria control needs further study. |
| Better understanding of the ecology, behaviour, and genetic population structure of malaria vectors, particularly outdoor biting and resting species that escape current vector control tools.  Development of innovative new technologies that can:  Educate the community effectively and engage the consumer market.  Control outdoor biting and resting mosquito vectors.  Simply and rapidly measure transmission. | The dominant Asia-Pacific *Anopheles* species were mapped.  Studies are showing the impact of tools on vector populations. The use of ITNs was linked to a decline of *A. gambiae* populations, but increased the proportion of insects that feed outdoor.  New methods are under investigation: sugar baits;, insecticide synergies;, exposure baseline numbers; surveying methodologies.  Use of spatial repellents in a large multi-centre trial is underway to evaluate the efficacy of one type of spatial repellent.  Research on importance of outdoor biting vectors and secondary vectors in areas with high coverage of nets and IRS has started but needs to be coordinated.  New tools are being developed for monitoring and evaluation of measure transmission but none ready for operational research.  See detailed narrative* | *Sinka, 2011; Bayoh, 2010; Reddy, 2011; Russell, 2011; Beier, 2012; Farenhorst, 2010; Huho, 2013; Chaki, 2012; Govella, 2011; Tusting, 2014; Achee.* | Here we need better monitoring of insect ecology and the development of innovative genetic and behaviour-altering tools.  There is a need for inexpensive, simple, and convenient to use molecular assays for mosquito speciation to replace morphology-based methods.  More robust vector control tools are needed directed at early biting and outdoor biting species. Moreover, they should be accessible and acceptable for the populations at risk.  We require a better understanding of how volatile organic compounds may act as spatial mosquito bite deterrents and improve our understanding of how they affect mosquito behaviour. |
| Sustained commitment to the long-term development of novel approaches like the genetic manipulation of natural vector populations that will permanently reduce the very high vectorial capacities of dominant malaria vectors in sub-Saharan Africa and some parts of Asia. | We have seen an expansion of the number of insect species (and variants) whose genomes have been sequenced (sixteen Anopheles genomes in early 2015).  The proof-of principle has been demonstration for homing endonuclease gene (HEGs) as a potential control strategy.  There has been progress with generating precisely defined genetic modifications. CRISPR technology shown to be very effective means to drive genetic changes in insects with application of this technology to mosquitoes expected in the future.  Genetic modification of certain bacteria in the mosquito microbiota (paratransgenesis) appear to reduce mosquito vectorial competence and novel approaches are being tested which could be relevant to malaria transmitting vectors (e.g. dengue field experiments using *Wolbachia* paratransgenesis.  See detailed narrative** | *Nolan, 2011; Bernardini, 2014; Neafsey, 2015.* | Approaches for population replacement rather than simply population reduction.  Further use CRISPR or alternative approaches, to drive foreign genes into mosquitoes for vector population replacement. |

**Detailed narrative and panellists’ comments:*

There is a need for research in the basic biological and discovery of *Anopheles* ecology. This is complicated by the morphological similarity of many closely related members of the relevant *Anopheles* species complexes. The ability to molecularly distinguish these species is challenging either due to technical or financial barriers. We are aware of discussions to operationalize tools to move lab based “*Anopheles* barcoding” into the field. This would help fill a data gap. This area was recently boosted by an expansion of the number of insect species (and variants) whose genomes have been entirely sequenced (sixteen *Anopheles* genomes in early 2015 [Neafsey, 2015]).

Current vector control tools only targets anthropophilic and endophagic mosquitoes, while the early biting, outdoor biting species are not targeted. To eliminate this residual malaria transmission, additional vector control tools will be needed. This gap in protection needs to be tackled. Research has begun to investigate the importance of outdoor biting vectors and secondary vectors in areas with high coverage of nets and IRS. However, this research is patchy and needs to be coordinated to obtain a clearer picture of the current situation. Also of note is the use of poisonous sugar baits [Beier, 2012; Qualls, 2015] and electric fences in some parts of the world. Perhaps most exciting is the use of spatial repellents to protect people from malaria mosquitoes. Currently a large multi-centre trial is underway to evaluate the efficacy of one type of spatial repellent. There is another research gap here to be filled, products like insecticide-treated uniforms and emanators worn on the belt, mosquito screening, etc are rarely evaluated by researchers; this seems a lost opportunity.

Larval source management is now recognized as a supplementary tool for malaria control. Larval control will act to control both outdoor and indoor populations of vectors. A large number of potentially larvicidal agents, especially of biological origin are being explored, *e.g*. [Azokou, 2013; Bansal, 2014; Castelino, 2014; Dohutia, 2015; Eze, 2014; Govindarajan, 2014a, 2014b; Govindarajan, 2013; Karunamoorthi, 2014; D. Kumar, 2014; K. R. Kumar, 2015; Manjari, 2014; Mozaffari, 2014; Panneerselvam, 2013; Sama, 2014; Santhosh, 2015; Sharma, 2014; Subarani, 2013; Suryawanshi, 2015; Velu, 2015; Z. Q. Wang, 2015].

New tools are being developed for monitoring transmission, including clay pots and resting boxes for outdoor resting mosquitoes, odour-baited traps and tent traps for indoor-resting traps, electric-grid traps and double-net traps for estimating human-biting collections and oviposition traps for collecting gravid female malaria mosquitoes [Lima, 2014; Menger, 2015; Nyasembe, 2014; Pombi, 2014; Vezenegho, 2014; Wagman, 2015]. However, in our opinion, none of these traps are ready for operational research and all require further development and testing in different habitats and with different vector species.

***Detailed narrative and panellists’ comments:*

A major breakthrough was the discovery that CRISPR technology can be used as a very effective genetic drive in insects. Proof of concept in Drosophila has been recently published [Gantz, 2015] and application of this technology to mosquitoes should be expected in the near future. The CRISPR technology shows great promise but would be for population reduction purposes. An important challenge will be to devise means, using CRISPR or alternative approaches, to drive foreign (antimalarial) genes into mosquito vector populations (population replacement).

Progress has been made on one front (homing endonucleases) with proof-of principle demonstration of a possible control strategy [Galizi, 2014]. This is a population reduction, and not population replacement approach. Using a different strategy, researchers have shown that genetic modification of certain bacteria from the mosquito microbiota (paratransgenesis) can lead to dramatic reduction of mosquito vectorial competence [S. Wang, 2012]. The use of entomopathogenic fungi to control insect populations has shown promise. It has the advantage of being effective for control of insecticide-resistant mosquito populations [Fang, 2011; Farenhorst, 2009].

Vaccines are not the only tool capable of interrupting malaria transmission in a sustainable way. Genetic modification of malaria vectors to reduce either their numbers or their capacity to transmit the malaria parasite, and thus provide long- lasting and inexpensive area-wide control of transmission, has long been a dream. This is now increasingly becoming feasible because of the increased ease of generating precisely defined genetic modifications [Bernardini, 2014], an expansion of the number of insect species (and variants) whose genomes have been entirely sequenced (sixteen Anopheles genomes in early 2015 [Neafsey, 2015]), and an improved understanding of vector biology (esp. ethology). The latter also opens the possibility to select for altered behaviour traits which usually depend on complex genetics but which are not necessarily slower to become fixed than insecticide resistance, which may come at a fitness cost. For example, LLINS will strongly select for evasive behaviours that prevent mosquitoes from being killed by the insecticide, or otherwise result in their failure to reproduce because of lack of access to human blood. However, LLINS combined with sugar traps [Qualls, 2015] steer mosquitoes into behaviour patterns that permit (actually may promote) their survival but do not favour Plasmodium’s. It is extremely likely that if such traps are effective they will lead to the rapid loss of the highly complex genetics that underlies the delicate ethology and physiology of feeding on human hosts. As was pointed out in a recent model for this type of approaches [Killeen, 2014b], it is desirable for this strategy that humans are the principal source of blood for these vector; for vectors that carry P. falciparum or P. vivax this appears indeed to be the case [Kiszewski, 2004].

As an alternative to driving population behavioural changes (where the genetics follows), increasingly precise methods now exists to directly/rationally modify insect genomes. Austin Burt's group provided the first successful demonstration of gene drive in A. gambiae mosquitoes in a model system using a yeast homing endonuclease in mosquitoes transgenic for its natural target [Windbichler, 2011]. If such a system can be modified to recognize genes involved in mosquito fertility or viability this could provide a long-lasting method for reducing their numbers. Modification of other characteristics also could decrease transmission, such as the ability to recognize human odours.

The Burt group has developed a method using homing endonuclease to bias the results of mating between transgenic and wild type mosquitoes to over 95% male offspring that, if it can successfully be combined with gene drive, could result in reduced numbers of mosquito vectors [Galizi, 2014]. If new results showing homing with CRISPR/Cas 9 nuclease constructs in Drosophila [Gantz, 2015] can be adapted to mosquitoes, this could result in a gene drive system that is easier to engineer than natural homing endonucleases, and several groups are working on this. The bacterium *Wolbachia* exhibits a form of gene drive based on cytoplasmic incompatibility, and release of trans-infected *Aedes aegypti* mosquitoes has been shown to establish the infection in local wild type mosquitoes [Hoffmann, 2011; Walker, 2011]. Some strains of *Wolbachia* inhibit replication of viral and other types of pathogens, including Plasmodium in mosquitoes [Ferguson, 2015], and *Wolbachia* has been proposed as a potential method to inhibit the vectorial capacity of the Asian malaria vector *Anopheles stephensi* [Bian, 2013]. It is, however, unclear whether this method would work in Africa because of the need to reach a rather high invasion threshold before the bacteria are able to establish and spread into the native population. Using a different strategy, researchers have shown that genetic modification of certain bacteria from the mosquito microbiota (paratransgenesis) can lead to dramatic reduction of mosquito vectorial competence [S. Wang, 2012]. The use of entomopathogenic fungi to control insect populations has also shown promise. It has the advantage of being effective for control of insecticide-resistant mosquito populations [Fang, 2011; Farenhorst, 2009].

It will be important to make a serious investment into approaches of population replacement (e.g. steering insects away from human blood to an alternative food source), as opposed to population reduction. This is because population reduction results in an "empty biological niche', which is readily filled with vector mosquitoes as soon as control measures become ineffective, are interrupted or are discontinued.
